# Genomic Features of High-Priority *Salmonella enterica* Serovars Circulating in the Food Production Chain, Brazil, 2000–2016

**DOI:** 10.1038/s41598-019-45838-0

**Published:** 2019-07-30

**Authors:** Daniel F. Monte, Nilton Lincopan, Hanna Berman, Louise Cerdeira, Shivaramu Keelara, Siddhartha Thakur, Paula J. Fedorka-Cray, Mariza Landgraf

**Affiliations:** 1 Department of Food and Experimental Nutrition, Faculty of Pharmaceutical Sciences, Food Research Center, University of São Paulo, São Paulo, Brazil; 2 Department of Microbiology, Institute of Biomedical Sciences, University of São Paulo, São Paulo, Brazil; 3 Department of Clinical Analysis, Faculty of Pharmaceutical Sciences, University of São Paulo, São Paulo, Brazil; 4 Department of Population Health and Pathobiology, North Carolina State University, College of Veterinary Medicine, Raleigh, North Carolina USA

**Keywords:** Comparative genomics, Antimicrobial resistance

## Abstract

Multidrug-resistant (MDR) *Salmonella enterica* has been deemed a high-priority pathogen by the World Health Organization. Two hundred and sixty-four *Salmonella enterica* isolates recovered over a 16-year period (2000 to 2016) from the poultry and swine production chains, in Brazil, were investigated by whole-genome sequencing (WGS). Most international lineages belonging to 28 serovars, including, *S*. *enterica* serovars *S*. Schwarzengrund ST96, *S*. Typhimurium ST19, *S*. Minnesota ST548, *S*. Infantis ST32, *S*. Heidelberg ST15, *S*. Newport ST45, *S*. Brandenburg ST65 and *S*. Kentucky ST198 displayed MDR and virulent genetic backgrounds. In this regard, resistome analysis revealed presence of *qnrE1* (identified for the first time in *S*. Typhimurium from food chain), *qnrB19, qnrS1*, *bla*_CTX-M-8_, *bla*_CTX-M-2_ and *bla*_CMY-2_ genes, as well as *gyrA* mutations; whereas ColpVC, IncHI2A, IncHI2, IncFIA, Incl1, IncA/C2, IncR, IncX1 and po111 plasmids were detected. In addition, phylogenetic analysis revealed multiple independent lineages such as *S*. *enterica* serovars *S*. Infantis, *S*. Schwarzengrund, *S*. Minnesota, *S*. Kentucky and *S*. Brandenburg. In brief, ocurrence and persistence of international lineages of *S*. *enterica* serovars in food production chain is supported by conserved genomes and wide virulome and resistome.

## Introduction

*Salmonella enterica* displaying resistance to fluoroquinolone and third-generation cephalosporin remains one of the most pressing global concerns, being deemed a high-priority pathogen by the World Health Organization (WHO)^1^. In this regard, quinolone efflux pumps (*oqxA*/*oqxB*) and plasmid-mediated quinolone resistance (PMQR) genes, such as *qnrB* and *qnrS* variants have contributed to the increase in fluoroquinolone resistance^2–4^. On the other hand, the coexistence of PMQR and extended-spectrum beta-lactamases (ESBLs), most CTX-M-type, in *S*. *enterica* isolates is being increasingly reported at the human-animal interface worldwide^4–6^.

Since, there is a growing understanding that the food production chain plays an important role both in the transmission of antibiotic-resistant pathogens and in their evolution and dissemination^4^. Genomic investigation of high-priority *S*. *enterica* serovars is a fundamental component of epidemiological surveillance work, particularly in major food-producing countries. Therefore, we have conducted a genomic investigation of *S*. *enterica* isolates recovered over a 16-year period from the poultry and swine meat production chains, in Brazil, which currently is one of the largest exporters of chicken and swine meat. In this regard, our results highlight persistence and dissemination of international lineages of multidrug-resistant *S*. *enterica* serovars exhibiting a highly virulent genetic background, in the food production chain.

## Materials and Methods

### Bacterial isolates

During a national surveillance study, 264 nontyphoidal *Salmonella enterica* (NTS) isolates recovered over a 16-year period (2000 to 2016), from the poultry and swine production chains, in Brazil, have been subjected to antimicrobial resistance screening. Specifically, in this study, we have focused on high-priority *S*. *enterica* isolates (n = 43) harboring fluoroquinolones, extended-spectrum β-lactamase (ESBL), and/or plasmid-mediated AmpC (pAmpC) resistance genes. Non-clinical samples were obtained from different points of poultry and swine production chain (Supplementary-Table 1) representing four different geographic regions in Brazil: South (Parana and Santa Catarina), Southeast (Sao Paulo and Minas Gerais), Midwest (Distrito Federal), and Northeast (Bahia) (Supplementary-Table 1). Bacterial isolation and serotyping were performed as previously reported^7–9^. All isolates (n = 43) underwent phenotypic and molecular characterization by Kirby-Bauer disc diffusion (Cefar), as well as broth microdilution using Sensititre® Gram Negative Plates (Trek Diagnostic Systems, OH) and whole-genome sequencing, respectively. Mininmum inhibitory concentration (MIC) values were interpreted according to Clinical and Laboratory Standards Institute (CLSI)^10^ and the National Antimicrobial Resistance Monitoring System (NARMS)^11^.

### Whole-Genome sequencing

DNA extraction was performed using a commercial kit (QiAmp tissue, Qiagen, Germany) according to manufacturer’s guidelines. Genomic DNA of *S*. *enterica* isolates (n = 43) harboring fluoroquinolones, ESBL and/or pAmpC resistance genes were sequenced at a 300-bp paired-end-read using the Nextera XT library preparation kit at the MiSeq platform (Illumina, San Diego, CA).

### Genomic data analysis

FastQ data were transferred into CLC genomic workbench 10.1.1 (Qiagen) and reads underwent strict quality control as previously described^12^. Subsequently, the software has ensured the appropriate size of the read length (300-bp), as well GC% content around 50%. Sequences obtained were *de novo* assembled using default settings in CLC workbench 10.1.1 (Qiagen). Resulting contigs were used to determine resistome (ResFinder 3.1), plasmidome (PlasmidFinder 2.0), multilocus sequence typing (MLST 2.0), plasmid MLST (pMLST 2.0), *Salmonella* pathogenicity island (SPIfinder 1.0), using default settings for all databases such as select threshold for %ID (≥90%) available in Center for Genomic Epidemiology (www.genomicepidemiology.org).

Assemblies were annotated with PROKKA version 1.14-dev^13^. The core genome was defined and aligned using Roary software version 3.11.2, with the BlastP threshold set to 95%^14^. The Roary software performs an all-against-all Blast of sequences produced by an initial CD-Hit clustering. The BlastP threshold was selected to include the mode of hits over a range of thresholds from 60 to 100%, while simultaneously providing a strict criterion to determine a set of vertically inherited core genes that can confidently be used to infer a phylogeny. A pan-genome gene presence-absence matrix was visualized with Phandango^15^ using the gene presence/absence output from Roary. Single nucleotide polymorphisms were extracted from the alignment using SNP-sites version 2.3.3^16^.

Phylogeny was reconstructed using RAxML version 8.2.12 with a General Time Reversible Model with Gamma distribution for rate heterogeneity^17^. The resulting phylogeny was tested against 600 bootstrap replications and the number of replications necessary was determined by implementing the majority rule, autoMR convergence criteria in the RAxML software^18^. The resulting phylogeny was visualized and annotated using iTol version 3^19^.

### Sequence data accession number

Sequence data were deposited as part of the GenomeTrakr Project: Thakur Molecular Epidemiology Laboratory, NC State University. We deposited all sequences (n = 43) in GenBank and their accession numbers are listed in Table 1.

**Table 1:** Features of *S*. *enterica* isolates (*n* = 43) harboring fluoroquinolones, extended-spectrum β-lactamase (ESBL), and/or plasmid-mediated AmpC (pAmpC) resistance genes with a wide virulome.

Strain ID	Serotype	Year	Location	Source	Resistance profile	PMQR	Resistance genes	Plasmid	Sequence type	Virulence genes
STy2 SAMN08606827	Typhimurium	2000	PR	Pig liver	CHL-TET-CIP-GEN-NAL-SXT-AMP-STR	*qnrE1*	*bla*_TEM-1B_, *aadA1*, *aac*(*3*)-*IIa*, *floR*, *sul1*, *tet* (*A*), *drfA1*	IncHI2A, IncHI2, IncFIA	ST3438	*invA*, *slyA*, *fimH*, *phoP*, *phoQ*, *sipABC*, *mgtA*
STy13 SAMN08874445	Typhimurium	2013	PR	Pork	CHL-TET-CIP-GEN-NAL-SXT-AMP-STR	*qnrE1*	*bla*_TEM-1B_, *aac*(*3*)-*IIa*, *aadA1*, *floR*, *sul1*, *tet* (*A*), *dfrA1*	IncHI2A, IncHI2, IncFIA	ST19	*invA*, *slyA*, *fimH*, *phoP*, *phoQ*, *sipABC*, *mgtA*
STy05 SAMN08386803	Typhimurium	2015	SP	Broiler chicken	TET-CIP-GEN-NAL-SXT-AMP-STR	*qnrE1*	*bla*_TEM-1B_, *aadA1*, *aac*(*3*)-*lla*, *aph*(*6*)-*ld*, *strA*, *sul1*, *tet*(*A*), *dfrA1*	IncHI2A, IncHI2, IncFIA	ST19	*invA*, *slyA*, *fimH*, *phoP*, *phoQ*, *sipABC*, *mgtA*
STy06 SAMN07279711	Typhimurium	2015	SP	Broiler chicken	CHL-TET-CIP-GEN-NAL-SXT-AMP-STR	*qnrE1*	*bla*_TEM-1B_, *strA*, *strB*, *aac*(*3*)-*IIa*, *aadA1*, *floR*, *sul1*, *tet* (*A*), *drfA1*	IncHI2A, IncHI2, IncFIA	ST19	*invA*, *slyA*, *fimH*, *phoP*, *phoQ*, *sipABC*, *mgtA*
STy07 SAMN08606818	Typhimurium	2015	SP	Broiler chicken	CHL-TET-CIP-GEN-NAL-SXT-AMP-STR	*qnrE1*	*bla*_TEM-1B_, *aac*(*3*)-*lla*, *aph*(*6*)-*ld*, *strA*, *aadA2*, *floR*, *sul1*, *tet*(*A*), *dfrA1*	IncHI2A, IncHI2, IncFIA	ST19	*invA*, *slyA*, *fimH*, *phoP*, *phoQ*, *sipABC*, *mgtA*
STy08 SAMN08874450	Typhimurium	2015	SP	Broiler chicken	CHL-TET-CIP-GEN-NAL-SXT-AMP-STR	*qnrE1*	*bla*_TEM-1B_, *aadA1*, *aac*(*3*)-*lla*, *floR*, *sul1*, *tet*(*A*), *dfrA1*	IncHI2A, IncHI2, IncFIA, ColpVC	ST19	*invA*, *slyA*, *fimH*, *phoP*, *phoQ*, *sipABC*, *mgtA*
STy011 SAMN08386758	Typhimurium	2015	SP	Broiler chicken	CHL-TET-CIP-GEN-NAL-SXT-AMP	*qnrE1*	*bla*_TEM-1B_, *aadA1*, *aac*(*3*)-*lla*, *floR*, *tet*(*A*)	IncHI2A IncHI2, IncFIA, po111	ST19	*invA*, *slyA*, *fimH*, *phoP*, *phoQ*, *sipABC*, *mgtA*
STy013 SAMN07283712	Typhimurium	2015	SP	Broiler chicken	CHL-TET-CIP-GEN-NAL-SXT-AMP	*qnrE1*	*bla*_TEM-1A_, *aadA1*, *aac*(*3*)-*IIa*, *sul1*, *tet* (*A*)	IncHI2A, IncHI2, IncFIA, Incl1	ST19	*invA*, *slyA*, *fimH*, *phoP*, *phoQ*, *sipABC*, *mgtA*
STy015 SAMN07279560	Typhimurium	2015	SP	Swine stomach	CHL-TET-CIP-NAL-AMP-STR	*oqxA*, *oqxB*,	*bla*_TEM-1A_ *strA*, *strB*, *floR*, *sul2*, *tet* (*B*)	IncR	ST19	*invA*, *slyA*, *fimH*, *phoP*, *phoQ*, *sipABC*, *mgtA*
SI017 SAMN08387036	Infantis	2015	SP	Swine muscle	CHL-TET-CIP-AMP-STR	*qnrS1*	*bla*_TEM-1B_, *aadA2*, *inu*(*F*),*floR*, *tet*(*A*)	IncR	ST32	*invA*, *slyA*, *fimH*, *phoP*, *phoQ*, *sipABC*, *mgtA*
SI018 SAMN08387035	Infantis	2015	SP	Swine muscle	CHL-TET-CIP-NAL-AMP-STR	*qnrS1*	*bla*_TEM-1B_, *aadA2*, *inu*(*F*),*floR*, *tet*(*A*)	IncR	ST32	*invA*, *slyA*, *fimH*, *phoP*, *phoQ*, *sipABC*, *mgtA*
SI690 SAMN08951109	Infantis	2016	SC	Chicken cage after cleaning	Pansusceptible	*qnrB19*	—	—	ST32	*invA*, *slyA*, *fimH*, *phoP*, *phoQ*, *sipABC*, *mgtA*
SSc117 SAMN08874439	Schwarzengrund	2016	SP	Swab	TET-AXO-CIP-GEN-NAL-XNL-SXT-AMP-STR	*qnrB19*	*bla*_CTX-M-2_, *aac*(*3*)-*IVa*, *strA*, *strB*, *aph*(*4*)-*Ia*, *aadA1*, *sul1*, *tet*(*A*), *tet*(*B*), *dfrA1*	IncHI2A, IncHI2, ColpVC	ST96	*invA*, *slyA*, *fimH*, *phoP*, *phoQ*, *sipABC*, *mgtA*
SSc119 SAMN08874447	Schwarzengrund	2016	SP	Mechanically recovered meat	TET-AXO-CIP-GEN-NAL-XNL-SXT-AMP-STR	*qnrB19*	*bla*_CTX-M-2_, *aac*(*3*)-*IVa*, *strA*, *strB*, *aph*(*4*)-*Ia*, *aadA1*, *sul1*, *tet*(*A*), *tet*(*B*), *dfrA1*	IncHI2A, IncHI2, ColpVC	ST96	*invA*, *slyA*, *fimH*, *phoP*, *phoQ*, *sipABC*, *mgtA*
SSc123 SAMN08874435	Schwarzengrund	2016	SP	Mechanically recovered meat	TET-AXO-CIP-GEN-NAL-XNL-SXT-AMP-STR	*qnrB19*	*bla*_CTX-M-2_, *aac*(*3*)-*IVa*, *strA*, *strB*, *aph*(*4*)-*Ia*, *aadA1*, *sul1*, *tet*(*A*), *tet*(*B*), *dfrA1*	IncHI2A, IncHI2, ColpVC	ST96	*invA*, *slyA*, *fimH*, *phoP*, *phoQ*, *sipABC*, *mgtA*
SSc126 SAMN08874434	Schwarzengrund	2016	SP	Chicken thigh	TET-AXO-CIP-GEN-NAL-XNL-SXT-AMP-STR	***qnrB19***	***bla***_**CTX-M-2**_, *aac*(*3*)-*IVa*, *strA*, *strB*, *aph*(*4*)-*Ia*, *aadA1*, *sul1*, *tet*(*A*), *tet*(*B*), *dfrA1*	IncHI2A, IncHI2, ColpVC	ST96	*invA*, *slyA*, *fimH*, *phoP*, *phoQ*, *sipABC*, *mgtA*
SSc130 SAMN08874436	Schwarzengrund	2016	SP	Chicken cage after cleaning	TET-AXO-CIP-GEN-NAL-XNL-SXT-AMP-STR	***qnrB19***	***bla***_**CTX-M-2**_, *aac*(*3*)-*IVa*, *strA*, *strB*, *aph*(*4*)-*Ia*, *aadA1*, *sul1*, *tet*(*A*), *tet*(*B*), *dfrA1*	IncHI2A, IncHI2, ColpVC	ST96	*invA*, *slyA*, *fimH*, *phoP*, *phoQ*, *sipABC*, *mgtA*
SSc140 SAMN08874410	Schwarzengrund	2016	DF	Chicken wing paddle	CIP-NAL	***qnrB19***	—	—	ST96	*invA*, *slyA*, *fimH*, *phoP*, *phoQ*, *sipABC*, *mgtA*
SSc146 SAMN08874411	Schwarzengrund	2016	DF	Chicken carcass	CIP-NAL	***qnrB19***	—	ColpVC	ST96	*invA*, *slyA*, *fimH*, *phoP*, *phoQ*, *sipABC*, *mgtA*
SSc149 SAMN08874405	Schwarzengrund	2016	DF	Chicken carcass	TET-CIP-GEN-NAL-SXT-STR	***qnrB19***	*aph*(*3’*)-*Ia*, *aac*(*3*)-*IVa*, *strA*, *strB*, *aph*(*4*)-*Ia*, *aadA1*, *sul1*, *tet*(*A*), *tet*(*B*), *dfrA1*	IncHI2A, IncHI2, ColpVC	ST96	*invA*, *slyA*, *fimH*, *phoP*, *phoQ*, *sipABC*, *mgtA*
SSc150 SAMN08874406	Schwarzengrund	2016	DF	Chicken carcass	TET-AXO-CIP-GEN-NAL-XNL-SXT-AMP-STR	***qnrB19***	***bla***_**CTX-M-2**_, *aph*(*3’*)-*Ia*, *aac*(*3*)-*IVa*, *strA*, *strB*, *aph*(*4*)-*Ia*, *aadA1*, *sul1*, *tet*(*A*), *tet*(*B*), *dfrA1*	IncHI2A, IncHI2, ColpVC	ST96	*invA*, *slyA*, *fimH*, *phoP*, *phoQ*, *sipABC*, *mgtA*
SSc151 SAMN08951138	Schwarzengrund	2016	DF	Chicken carcass	TET-AXO-CIP-GEN-NAL-XNL-SXT-AMP-STR	***qnrB19***	***bla***_**CTX-M-2**,_ *aph*(*3’*)-*Ia*, *aac*(*3*)-*IVa*, *strA*, *strB*, *aph*(*4*)-*Ia*, *aadA1*, *sul1*, *tet*(*A*), *tet*(*B*), *dfrA1*	IncHI2A, IncHI2, ColpVC	ST96	*invA*, *slyA*, *fimH*, *phoP*, *phoQ*, *sipABC*, *mgtA*
SSc156 SAMN08951134	Schwarzengrund	2016	DF	Chicken carcass	TET-AXO-CIP-GEN-NAL-XNL-SXT-AMP-STR	***qnrB19***	***bla***_**CTX-M-2**_, *aph*(*3’*)-*Ia*, *aac*(*3*)-*IVa*, *strA*, *strB*, *aph*(*4*)-*Ia*, *aadA1*, *sul1*, *tet*(*A*), *tet*(*B*), *dfrA1*	IncHI2A, IncHI2, ColpVC	ST96	*invA*, *slyA*, *fimH*, *phoP*, *phoQ*, *sipABC*, *mgtA*
SSc161 SAMN08951135	Schwarzengrund	2016	MG	Mechanically recovered meat	CIP-NAL	***qnrB19***	—	ColpVC	ST96	*invA*, *slyA*, *fimH*, *phoP*, *phoQ*, *sipABC*, *mgtA*
SN141 SAMN08951153	Schwarzengrund	2016	DF	Chicken carcass	CIP-NAL	***qnrB19***	—	ColpVC	ST96	*invA*, *slyA*, *fimH*, *phoP*, *phoQ*, *sipABC*, *mgtA*
SN143 SAMN08951160	Schwarzengrund	2016	DF	Chicken carcass	TET-AXO-CIP-GEN-NAL-XNL-SXT-AMP-STR	***qnrB19***	***bla***_**CTX-M-2**,_ *aph*(*3’*)-*Ia*, *aac*(*3*)-*IVa*, *strA*, *strB*, *aph*(*4*)-*Ia*, *aadA1*, *sul1*, *tet*(*A*), *tet*(*B*), *dfrA1*	IncHI2A, IncHI2, ColpVC	ST96	*invA*, *slyA*, *fimH*, *phoP*, *phoQ*, *sipABC*, *mgtA*
SN145 SAMN08951155	Schwarzengrund	2016	DF	Chicken carcass	TET-AXO-CIP-GEN-NAL-XNL-SXT-AMP-STR	***qnrB19***	***bla***_**CTX-M-2**_, *aph*(*3’*)-*Ia*, *aac*(*3*)-*IVa*, *strA*, *strB*, *aph*(*4*)-*Ia*, *aadA1*, *sul1*, *tet*(*A*), *tet*(*B*), *dfrA1*	IncHI2A, IncHI2, ColpVC	ST96	*invA*, *slyA*, *fimH*, *phoP*, *phoQ*, *sipABC*, *mgtA*
SMi132 SAMN08951176	Schwarzengrund	2016	SP	Chicken carcass	TET-AXO-CIP-GEN-NAL-XNL-SXT-AMP-STR	***qnrB19***	***bla***_**CTX-M-2**_, *aac*(*3*)-*IVa*, *aph*(*4*)-*Ia*, *aadA1*, *strA*, *strB*, *sul1*, *tet*(*A*), *tet*(*B*), *dfrA1*	IncHI2A, IncHI2, ColpVC	ST96	*invA*, *slyA*, *fimH*, *phoP*, *phoQ*, *sipABC*, *mgtA*
SMi152 SAMN08951180	Schwarzengrund	2016	DF	Chicken carcass	TET-AXO-CIP-GEN-NAL-XNL-SXT-AMP-STR	***qnrB19***	***bla***_**CTX-M-2**_, *aac*(*3*)-*IVa*, *aph*(*4*)-*Ia*, *aadA1*, *strA*, *strB*, *sul1*, *tet*(*A*), *tet*(*B*), *dfrA1*	IncHI2A, IncHI2, ColpVC	ST96	*invA*, *slyA*, *fimH*, *phoP*, *phoQ*, *sipABC*, *mgtA*
SH147 SAMN08951096	Schwarzengrund	2016	DF	Chicken carcass	TET-AXO-CIP-GEN-NAL-XNL-SXT-AMP-STR	***qnrB19***	***bla***_**CTX-M-2**_, *aac*(*3*)-*IV*, *aph*(*6*)-*Id*, *aadA1*, *aph*(*3''*)-*Ib*, *aph*(*3'*)-*Ia*, *aph*(*4*)-*Ia*, *sul1*, *tet*(*A*), *tet*(*B*), *dfrA1*, *qacEdelta1*	IncHI2A, IncHI2, ColpVC	ST96	*invA*, *slyA*, *fimH*, *phoP*, *phoQ*, *sipABC*, *mgtA*
SH154 SAMN08951103	Schwarzengrund	2016	DF	Chicken carcass	TET-AXO-CIP-GEN-NAL-XNL-SXT-AMP-STR	***qnrB19***	***bla***_**CTX-M-8**_, ***bla***_**CTX-M-2**_, *aadA15*, *aph*(*3'*)-*Ic*, *aph*(*4*)-*Ia*, *strA*, *strB*, *sul1*, *tet*(*A*), *tet*(*B*), *lnu*(*A*), *qacEdelta1*	IncHI2A, IncHI2, ColpVC, Incl1	ST96	*invA*, *slyA*, *fimH*, *phoP*, *phoQ*, *sipABC*, *mgtA*
SH157 SAMN08951105	Schwarzengrund	2016	DF	Chicken carcass	TET-AXO-CIP-GEN-NAL-XNL-SXT-AMP-STR	***qnrB19***	***bla***_**CTX-M-2**_, *aac*(*3*)-*IV*, *aph*(*6*)-*Id*, *aadA1*, *aph*(*3''*)-*Ib*, *aph*(*4*)-*Ia*, *sul1*, *tet*(*A*), *tet*(*B*), *dfrA1*, *qacEdelta1*	IncHI2A, IncHI2, ColpVC, Incl1	ST96	*invA*, *slyA*, *fimH*, *phoP*, *phoQ*, *sipABC*, *mgtA*
SMi124 SAMN08951178	Minnesota	2016	SP	Chicken carcass	FOX-TET-AXO-AUG2-CIP-NAL-XNL-AMP-STR	***qnrB19***	***bla***_**CTX-M-8**_, ***bla***_**CMY-2**_, *aadA1*, *sul2*, *tet*(*A*)	IncA/C2	ST548	*invA*, *slyA*, *fimH*, *phoP*, *phoQ*, *sipABC*, *mgtA*
SMi160 SAMN08951185	Minnesota	2016	MG	Chicken feet	FOX-TET-AXO-AUG2-CIP-NAL-XNL-AMP-STR	***qnrB19***	***bla***_***CMY*-*2***_, *sul2*, *tet* (*A*), *aadA1*, *aph*(*3'*)*Ia*	IncA/C2	ST548	*invA*, *slyA*, *fimH*, *phoP*, *phoQ*, *sipABC*, *mgtA*
SMi294 SAMN08951202	Minnesota	2016	SP	Mechanically recovered meat	Pansusceptible	***qnrB5***	*aac*(*6'*)-*Iaa*	ColpVC	ST548	*invA*, *slyA*, *fimH*, *phoP*, *phoQ*, *sipABC*, *mgtA*
SSc153 SAMN08951101	Minnesota	2016	DF	Chicken carcass	FOX-TET-AXO-AUG2-CIP-NAL-XNL-AMP-STR	***qnrB19***	***bla***_***CMY*-*2***_, *sul2*, *tet* (*A*), *aadA1*, *aph*(*3'*)*Ia*	IncA/C2	ST548	*invA*, *slyA*, *fimH*, *phoP*, *phoQ*, *sipABC*, *mgtA*
SK497 SAMN08951172	Kentucky	2016	BA	Chicken liver	Pansusceptible	***qnrB19***	*aac*(*6'*)-*Iaa*	ColpVC	ST198	*invA*, *slyA*, *fimH*, *phoP*, *phoQ*, *sipABC*, *mgtA*
SSc142 SAMN08874409	Newport	2016	DF	Chicken carcass	AXO-CIP-GEN-NAL-XNL-AMP-STR	***qnrB19***	***bla***_**CTX-M-2**_, *aac*(*3*)-*VIa*, *aadA1*, *sul1*	ColpVC	ST45	*invA*, *slyA*, *fimH*, *phoP*, *phoQ*, *sipABC*, *mgtA*
SN144 SAMN08951147	Newport	2016	DF	Chicken carcass	AXO-CIP-GEN-NAL-XNL-AMP-STR	***qnrB19***	***bla***_**CTX-M-2**_, *aac*(*3*)-*VIa*, *aadA1*, *sul1*	—	ST45	*invA*, *slyA*, *fimH*, *phoP*, *phoQ*, *sipABC*, *mgtA*
SH291 SAMN09207939	Brandenburg	2016	SP	Chicken breast	FOX-TET-AXO-AUG2-CIP-NAL-XNL-AMP	***qnrB19***	***bla***_**CMY-2**_, *fosA7*, *sul2*, *tet* (*A*)	ColpVC, Incl1	ST65	*invA*, *slyA*, *fimH*, *phoP*, *phoQ*, *sipABC*, *mgtA*
SH686 SAMN09207883	Brandenburg	2016	SC	Mechanically recovered meat	TET-AXO-AUG2-CIP-NAL-XNL-AMP	***qnrB19***	*tet*(*A*)	ColpVC	ST65	*invA*, *slyA*, *fimH*, *phoP*, *phoQ*, *sipABC*, *mgtA*
SH159 SAMN08951099	Heidelberg	2016	MG	Chicken cage after cleaning	FOX-TET-AXO-AUG2-CIP-NAL-XNL-AMP	***qnrB5***	***bla***_**CMY-2**_, *fosA7*, *sul2*, *tet* (*A*), *aac*(*6'*)-*Iaa*	ColpVC, IncA/C2, Incl1, IncX1	ST15	*invA*, *slyA*, *fimH*, *phoP*, *phoQ*, *sipABC*, *mgtA*
SSc139 SAMN08874407	Heidelberg	2016	SP	Chicken wing	FOX-TET-AXO-AUG2-CIP-NAL-XNL-AMP-STR	***qnrB19***	***bla***_**CMY-2**_, *fosA7*, *sul2*, *tet*(*A*)	ColpVC, IncA/C2, Incl1, IncX1	ST15	*invA*, *slyA*, *fimH*, *phoP*, *phoQ*, *sipABC*, *mgtA*

## Results

### *Salmonella* isolates and distribution of serotypes

Amongst 264 *S*. *enterica* isolates, we identified 28 serotypes distributed in the six regions studied (Supplementary-Table 1). Most isolates included *S*. Heidelberg (n = 81), *S*. Typhimurium (n = 43), *S*. Infantis (n = 35), *S*. Schwarzengrund (n = 21), and *S*. Enteritidis (n = 20). The remaining sixty-four isolates were classified in 23 different serotypes, including rarely reported *S*. Abony (n = 6), *S*. Isangi (n = 4), *S*. Rochdale (n = 3), *S*. Saphra (n = 2), *S*. Orion (n = 2) *S*. Ouakam (n = 1), *S*. Grumpensis (n = 1), *S*. Carrau (n = 1), *S*. Abaetetuba (n = 1), and *S*. Idikan (n = 1) (Supplementary-Table 1).

In this study, 264 isolates were screened for resistance to fluoroquinolones and/or broad-spectrum cephalosporins, of which 40 isolates were considered as high priority *Salmonella* strains due to broad-spectrum cephalosporin- or fluoroquinolone-resistance profiles^1^. Then, genomic investigation was performed on 43 isolates, including high priority *Salmonella* strains displaying an MDR (n = 36), defined as resistant to three or more classes of antimicrobial compounds^20^; or a quinolone-resistant profile (n = 4). Additionally, 3 representative pan-susceptible *Salmonella* serovars *S*. Infantis (SI690), *S*. Minnesota (SMi294) and *S*. Kentucky (SK497) were also investigated by WGS, for comparative analysis (Table 1).

**Figure 1:**
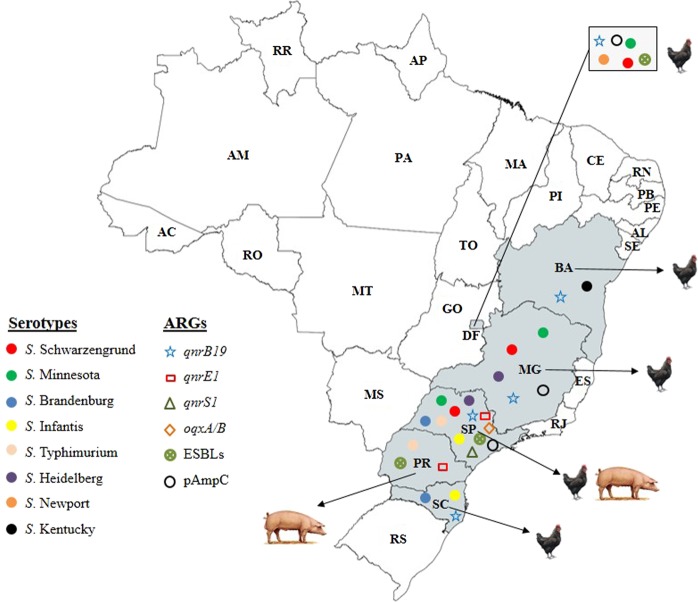
Distribution of *S*. *enterica* isolates (n = 43) harboring fluoroquinolones, extended-spectrum β-lactamase (ESBL), and/or plasmid-mediated AmpC (pAmpC) resistance genes and antimicrobial resistance genes (ARGs) over a 16-year period in Brazil. The map showing the distribution of *S. enterica* (n = 43) was created using an online service (https://mapchart.net/). Footnotes: *DF, Distrito Federal; MG, Minas Gerais; SP, São Paulo; PR, Paraná; SC, Santa Catarina; BA, Bahia.

Moreover, of the six Brazilian states studied, Sao Paulo (SP) harbored nineteen *S*. *enterica* isolates beloging to different serovars such as *S*. Typhimurium (n = 7), *S*. Schwarzengrund (n = 6), *S*. Infantis (n = 2), *S*. Minnesota (n = 2), *S*. Brandenburg (n = 1) and *S*. Heidelberg (n = 1). *Salmonella* Infantis (n = 1) and *S*. Brandenburg (n = 1) were isolated from the Santa Catarina (SC) state while the Distrito Federal (DF) harbored *S*. Schwarzengrund (n = 13), *S*. Newport (n = 2) and *S*. Minnesota (n = 1). Finally, the state of Minas Gerais (MG) harbored serotypes *S*. Schwarzengrund (n = 1), *S*. Minnesota (n = 1), and *S*. Heidelberg (n = 1), followed by Parana (PR) and Bahia (BA) with occurrence of *S*. Typhimurium (n = 2) and *S*. Kentucky (n = 1), respectively as summarized in Table 1 and Fig. 1.

### Resistome

While these 43 *Salmonella* isolates reimaned susceptible to colistin and carbapenems, additional genes encoding resistance to aminoglycoside [*aadA1, aadA2, aac*(*6*′)*, aac*(*3*)-*Iva, aph*(*4*)-*Ia, aac*(*3*)-*IIa*], sulfonamide [*sul1, sul2*], tetracycline [*tet*(*A*)*, tet*(*B*)], trimethoprim [*drfA1*], phenicol [*floR*], streptomycin [*strA, strB*], fosfomycin [*fosA7*], macrolide [*inu*(*F*)], and quaternary ammonium [*qacEdelta1*] were confirmed by WGS as shown in Table 1. All of these genes have been previously reported in *Salmonella* isolates from variety sources including food and human^5,21^.

Among 43 selected isolates, which presented genes conferring resistance to fluoroquinolones, ESBLs and/or pAmpC, 36 carried both PMQR and β-lactams genes, whereas 7 isolates presented PMQR but not β-lactams encoding genes. These isolates belonged to eight serovars including *S*. Schwarzengrund (n = 20), *S*. Typhimurium (n = 9), *S*. Minnesota (n = 4), *S*. Infantis (n = 3), *S*. Heidelberg (n = 2), *S*. Newport (n = 2), *S*. Brandenburg (n = 2) and *S*. Kentucky (n = 1).

The PMQR *qnrB19* gene, (n = 32) [8 isolates were only positive for *qnrB19*, and 24 co-produced CTX-M-2, CTX-M-8 or CMY-2 genes] was the most commom quinolone resistance gene observed followed by *qnrE1* (n = 8) [1 was positive for TEM-1A and 7 co-produced TEM-1B], *qnrS1* (n = 2) [both co-produced TEM-1B], and *oqxA*/*oqxB* (n = 1) [co-produced TEM-1A].

The highest PMQR gene diversity was observed in strains isolated from samples collected in Sao Paulo which harbored *qnrE1* (n = 6), *qnrB19* (n = 9), *qnrS1* (n = 2) and *oqxA/B* (n = 1). Subsequently, Distrito Federal [n = 16; (*qnrB19*)], Minas Gerais [n = 3; (*qnrB19*)], Santa Catarina [n = 2; (*qnrB19*)], Parana [n = 2; (*qnrE1*) and Bahia [n = 1; (*qnrB19*)] harbored different genes. The ESBL/pAampC distribution is shown in Table 1. Among these 43 isolates the most frequent source of *S*. *enterica* was poultry (34/43; 79%), followed by swine (5/43; 11.6%) and different sources (4/43; 9.3%), including chicken cage after cleaning (n = 3) and swab (n = 1) (Table 1).

**Figure 2:**
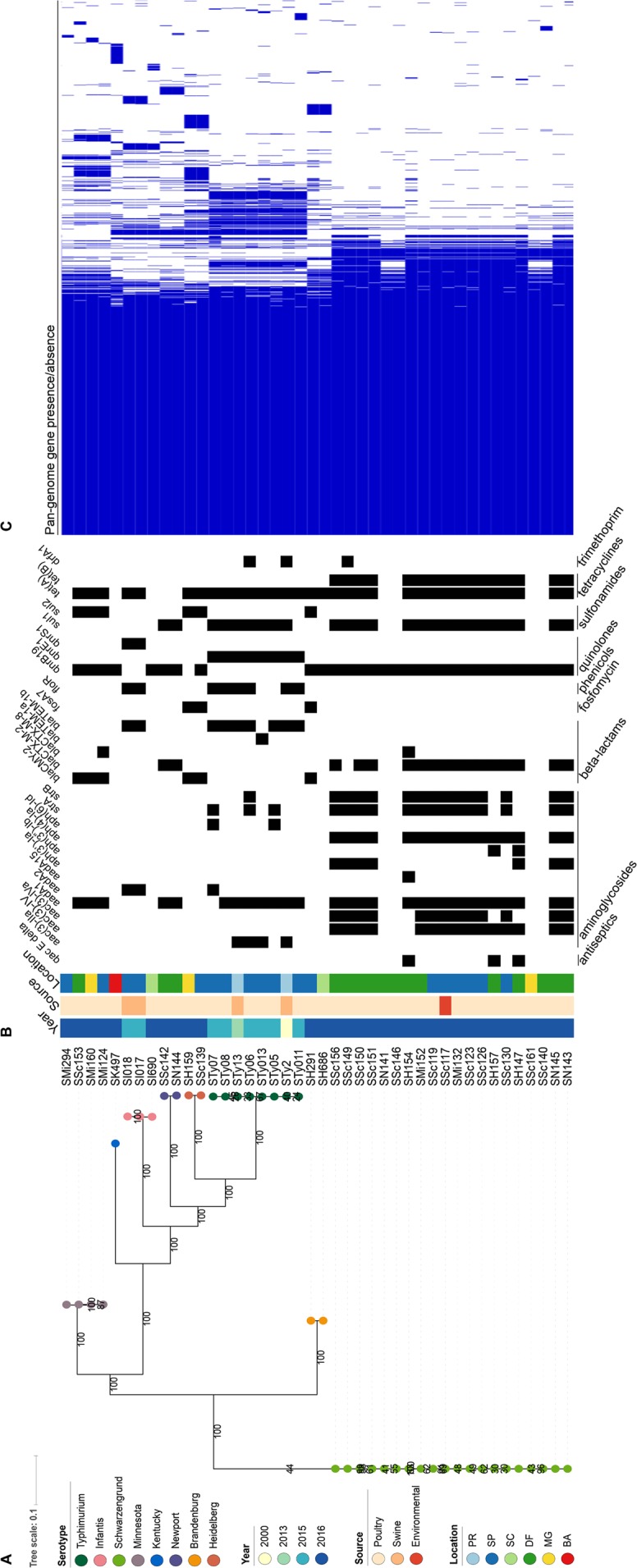
(**A**) Reconstructed phylogeny based on the core genome (3533 genes) of the 42 *Salmonella* strains. Percent of bootstrap samples in which nodes appeared are shown. The serotype of each isolate is labeled on its respective branch. Color strips depict the year, source, and geographic location of isolation, respectively. Poultry sources include broiler chicken, chicken wing, chicken wing paddle, chicken thigh, chicken feet, chicken breast, chicken cage after cleaning, chicken carcass, and Mechanically recovered chicken meat. Swine sources include: swine stomach, liver, muscle, and pork. Environmental sources include slaughterhouse and swab. (**B**) Presence and absence of selected antimicrobial resistance genes are shown, black indicating presence. (**C**) The gene presence/absence matrix depicts pan-genome variation.

In addition to overall resistance, the MIC results reveals 13 resistance patterns among these *S*. *enterica* isolates (n = 43) (Table 1). The MIC values ranged from 0.5 µg/mL to 512 µg/mL among 14 antibiotics tested. The majority of quinolone-resistant phenotypes (QRP) isolates presented high-level resistance (MIC ≥32 µg/mL) to nalidixic acid and range between 0.5 to 8 µg/mL for ciprofloxacin. Additionally, most QRP isolates were resistant to levofloxacin (third-generation quinolone), moxifloxacin (fourth generation quinolone) and enrofloxacin (veterinary use only) by using Kirby-Bauer disc diffusion method. Regarding broad-spectrum cephalosporin-resistant, all isolates harbouring *bla*_CTX-M_-type presented high-level resistance against ceftriaxone (MIC >64 µg/ml) and ceftiofur (>8 µg/ml). Besides ceftriaxone and ceftiofur (veterinary use only), the isolates that carried *bla*_CMY-2_ had high-level resistance against cefoxitin (>32 µg/ml). Further, fourty isolates were resistant to ciprofloxacin and interestingly, one isolate (*S*. Infantis) was ciprofloxacin-resistant but nalidixic acid susceptible. Lastly, a total of 33 genes in this collection encoded resistance to β-lactams, aminoglicosydes, sulphonamides, tetracycline, phenicols, trimethoprim, microlides, fosfomycin and amonium quaternary (Fig. 2).

### Quinolone resistance-determining region (QRDR) among *Salmonella enterica* isolates

Thirteen isolates (30.2%) exhibited a single mutation in *gyrA* at codons Ser83 and Asp87, which has been most frequently reported in *S*. *enterica*^22^. Of these, eight *S*. Typhimurium presented mutation at codon Ser83Tyr, one *S*. Typhimurium isolate at codon Asp87Asn, two *S*. Brandenburg isolates at codon Asp87Gly, and two *S*. Heidelberg isolates at codon Ser83Phe. While *gyrB*, *parC* and *parE* were not identified, this single mutation in *gyrA* was sufficient to promote resistance at >32 µg/mL and >4 µg/mL for nalidixic acid and ciprofloxacin, respectively; it was particularly observed among *S*. Typhimurium isolates carrying *qnrE1* and *gyrA* mutation, as shown in Table 2. Notably, the isolates, which harbored the combination between *gyrA* mutations and *qnrE1*, played a markedly greater role than *gyrA* and *qnrB19* in mediating quinolone resistance (Table 2).

**Table 2:** Co-occurrence of QRDR and PMQR among *S*. *enterica* serovars.

*S. enterica* strain	Serotype	Source	Date (year)	States^a^	MIC (µg/mL)^b^		Quinolone resistance-determining region (QRDR)			PMQR
					NAL	CIP	Nucleotide change	Amino acid change	Gene	
STy2	Typhimurium	Pig liver	2000	PR	>32	>4	TCC → TAC	Ser83Tyr	*gyrA*	*qnrE1*
STy13	Typhimurium	Pork	2013	PR	>32	>4	TCC → TAC	Ser83Tyr	*gyrA*	*qnrE1*
STy05	Typhimurium	Broiler chicken	2015	SP	>32	>4	TCC → TAC	Ser83Tyr	*gyrA*	*qnrE1*
STy06	Typhimurium	Broiler chicken	2015	SP	>32	>4	TCC → TAC	Ser83Tyr	*gyrA*	*qnrE1*
STy07	Typhimurium	Broiler chicken	2015	SP	>32	>4	TCC → TAC	Ser83Tyr	*gyrA*	*qnrE1*
STy08	Typhimurium	Broiler chicken	2015	SP	>32	>4	TCC → TAC	Ser83Tyr	*gyrA*	*qnrE1*
STy011	Typhimurium	Broiler chicken	2015	SP	>32	>4	TCC → TAC	Ser83Tyr	*gyrA*	*qnrE1*
STy013	Typhimurium	Broiler chicken	2015	SP	>32	>4	TCC → TAC	Ser83Tyr	*gyrA*	*qnrE1*
STy015	Typhimurium	Swine stomach	2015	SP	>32	>0.5	GAC → AAC	Asp87Asn	*gyrA*	*oqxA/oqxB*
SH291	Brandenburg	Chicken breast	2016	SP	>32	>1	GAC → GGC	Asp87Gly	*gyrA*	*qnrB19*
SH686	Brandenburg	Mechanically recovered chicken meat	2016	SC	>32	>1	GAC → GGC	Asp87Gly	*gyrA*	*qnrB19*
SH159	Heidelberg	Chicken cage after cleaning	2016	MG	>32	>0.25	TCC → TTC	Ser83Phe	*gyrA*	*qnrB19*
SSc139	Heidelberg	Chicken wing	2016	SP	>32	>1	TCC → TTC	Ser83Phe	*gyrA*	*qnrB19*

^a^PR: Parana; SP: Sao Paulo; SC: Santa Catarina; MG: Minas Gerais;

^b^NAL: nalidixic acid; CIP: ciprofloxacin; MIC: minimum inhibitory concentration^a^.

### Identification of international lineages among fluoroquinolone- and cephalosporin-resistant *Salmonella enterica* serovars

We obtained among 43 *S*. *enterica* isolates a total of 9 different sequence types (STs) including the most frequently observed ST96 [*S*. Schwarzengrund (n = 20)], ST19 [*S*. Typhimurium (n = 8)] and ST548 [*S*. Minnesota (n = 4)]. As described above for *S*. Schwarzengrund, *S*. Typhimurium, and *S*. Minnesota, all STs were consistently associated with their respective serotypes: *S*. Infantis [ST32 (n = 3; 100%)], *S*. Heidelberg [ST15 (n = 2; 100%)], *S*. Newport [ST45 (n = 2; 100%)], *S*. Brandenburg [ST65 (n = 2; 100%)], and also the clinically important ST198 [*S*. Kentucky (n = 1; 100%)]. Only the *S*. Typhimurium contained two sequence types, being ST19 (n = 8) and ST3438 (n = 1), highlighting their genetic diversity.

### Mobilome

The most common plasmid incompatibility group in our collection was ColpVC (n = 27; 62%). Furthermore, a range of plasmids previously associated with multidrug-resistant foodborne bacteria which have been associated with clinical settings harbored: IncHI2A (n = 24; 55.8%), IncHI2 (n = 24; 55.8%), IncFIA (n = 8; 18.6%), Incl1 (n = 6; 14%), IncA/C2 (n = 5; 11.6%), IncR (n = 3; 7%), IncX1 (n = 2; 4.6%), po111 (n = 1; 2.3%) (Table 1).

**Figure 3:**
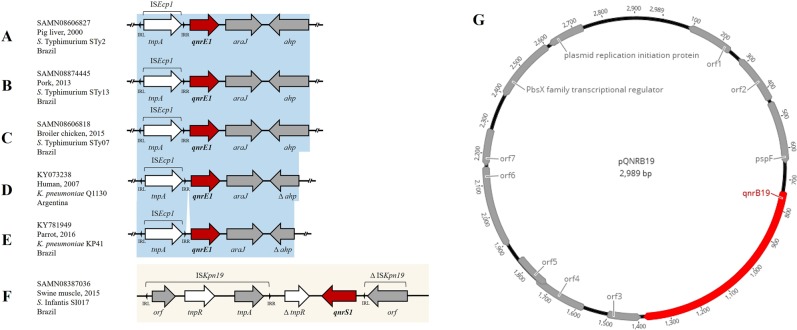
(**A**–**E**) Comparison of the genetic environments of *qnrE1* gene. (**F**) Genetic environment of *qnrS1*. (**G**) Representative *qnrB19* plasmid for 29 *S*. *enterica* isolates. Genes, different plasmids and shotgun sequences were extracted from GenBank database. Arrows indicate the positions and directions of the genes; Δ indicates the truncated gene. Regions with >99% identity are indicated in blue shadow.

The eight *S*. Typhimurium *qnrE1*-positive isolates exhibited identical genetic content, regardless of the source (swine or chicken) or year of isolation (2000, 2013, 2015) (Fig. 3). These plasmids were composed by IRL (inverted repeat left)-IS*Ecp1*-IRR (inverted repeat right)-*qnrE1*-*araJ*-*ahp* in a total of 4,659-bp and were different from previous reports^23,24^ (Fig. 3).

Twenty-nine *S*. *enterica* containing *qnrB19* genes had the plasmid sequences closed at 2,989-bp and each plasmid shared the same incompatibility group, ColpVC (Fig. 3). These small plasmids were identical to *qnrB19* that were identified in *E*. *coli* in Brazil in 2016 (KX452393.1). They also shared 99% of identity with *qnrB19*-plasmids from *Salmonella* Muenchen in the United States in 2017 (KY991368.1) and from *S*. *enterica* serovars in Canada in 2018 (CP030230.1). The remaining three isolates had large plasmids ranging from 3,159-bp (*S*. Schwarzengrund) to 37,696-bp (*S*. Heidelberg). Notably, the *qnrB19*-backbone (37,696-bp) of *S*. Heidelberg (SH159) isolated from a chicken cage after cleaning in this study, showed 100% identity with *S*. Heidelberg previously reported from human, animal and food sources in Canada in 2016 (CP016580.1) denoting intercontinental spread of bacteria harboring these genes.

The genetic context of *qnrS1* on *S*. Infantis had approximately 4,755-bp and this gene was surrounded upstream by truncated *tnpR* and downstream by truncated ISK*pn19* that were carried on a core quinolone resistance genetic enviroment (Fig. 3). Additionally, the genetic context of *bla*_CTX-M-8_ showed that this gene was flanked by two copies of IS*26*; one which was located 1,922-bp upstream followed by the transposase (*tnpA*) and IS*26* located 869-bp downstream for a total of 3,668-bp (IS*26*-*tnpA*-*bla*_CTX-M-8_-IS*26*). These data indicated that this genetic platform has the same mobilization apparatus of CTX-M-8-producing *E*. *coli*, which has been primarily isolated from retail chicken meat imported from Brazil^25^. Lastly, IncA/C2 was associated with *sul2*/*tetA* in *S*. Heidelberg, while IncHI2 carried *bla*_CTX-M-2_ and Incl1 was responsible by dissemination of *bla*_CMY-2_.

### Virulome and *Salmonella* pathogenicity Island

All 43 *Salmonella* genomes were analyzed for virulence factors using Prokka anotation and SPIfinder 1.0 (https://cge.cbs.dtu.dk/services/SPIFinder/). Several virulence factors were shared between *S*. *enterica* serotypes. All isolates were positive for *inv*A and *sly*A genes, which are responsible for host invasion and cytolysin production, respectively. Most *S*. *enterica* displayed important virulence factors involved in pathogenicity process. For example, *fimH* (n = 43) is an adhesin responsible for host cell specific recognition^26^. Further, were detected in all isolates components of *Salmonella* Pathogenicity Island (SPI) composed of virulence genes such as *phoP*, *phoQ*, *pagP*, *sipA*, *sipB*, *sipC*, and *mgtA*. The genes *phoP* and *phoQ* are responsible for the control of *HilE* expression which in turn regulates the expression of SPI-1^27^. Additionally, the others virulence genes (*pagP*, *sipABC*, *mgtA*) are involved in modification of lipid A (intracelular survival and ions transport), mechanisms not only involved in pathogenicity, but also in AMR^28^.

*Salmonella* Pathogenicity Islands harbor a variety of virulence genes, of which most are chromosomally located. These genes are required for interaction among *Salmonella* spp. and hosts^29^. Most of them are integrated with SPIs and the majority of the *Salmonella* isolates possess SPI-1^29^. However, the absence or partial deletions of these genetic components in certain circumstances does not interfere in the ability to cause disease, remaining potentially pathogenic presenting infection process such as invasion, intracellular survival and replication^29^. In fact, our findings revealed that not all serovars harbor SPI-1 as shown in Table 3. In this regard, a limitation of this study was the lack of an *in vivo* analysis for confirmation of the virulence behavior. However, our results confirm previous reports of association between SPI and serotype^30^ and highlight risk factors associated with *Salmonella* host infection^21^.

**Table 3:** Representative distribution of *Salmonella* Pathogenicity Island among *Salmonella* serovars.

*S*. *enterica* Serovars	*Salmonella* Pathogenicity Island^a^
*S*. Typhimurium	SPI-1, SPI-2, SPI-3, SPI-5, SPI-9, SPI-13, SPI-14, C63PI
*S*. Infantis	SPI-2, SPI-3, SPI-4, SPI-5, SPI-13, SPI-14, C63PI
*S*. Heidelberg	SPI-2, SPI-3, SPI-5, SPI-13, SPI-14, C63PI
*S*. Newport	SPI-3, SPI-4, SPI-5, SPI-13, SPI-14, C63PI
*S*. Schwarzengrund	SPI-3, SPI-4, SPI-13, SPI-14, C63PI
*S*. Brandenburg	SPI-2, SPI-3, SPI-4, SPI-13, C63PI
*S*. Minnesota	SPI-2, SPI-3, SPI-13, SPI-14, C63PI
*S*. Kentucky	SPI-2, SPI-3, SPI-4,
^a^C63PI: Centisome 63 pathogenicity island	

Interestingly, regarding virulome, we identified IS200 only among *S*. Brandenburg isolates. This isertion sequence was recently described in *S*. Typhimurium to be involved in host gene expression^31,32^.

### Phylogenetic and evolutionary dinamics of *S*. *enterica* isolates

The core genome used for phylogeny reconstruction represented a sizeable portion of the pan-genome, 3533 out of 8286 total genes in the pan-genome (Fig. 2). Each serotype was represented by a monophyletic clade on the reconstructed phylogeny, and bootstrap values of these clades were greater than 99, representing a high confidence in the phylogenetic topology. Genomic variation was observed between serovars, and specifically, antimicrobial resistance genes varied by serovar. The quinolone resistance gene allele was largely similar within serovar and varied between serovars (Fig. 2). Isolates did not cluster by year, source, or geographic location across the phylogeny.

The investigation of genomic diversity between *Salmonella* isolates is useful from an epidemiological perspective. We observed that specifically in *S*. *enterica* serotype and sequence type are the main drivers for cluster analysis, as most of the time isolates were clustered together by serotype and not by resistance profile, year, source or geographic location. Additionally, our results reveal high similarity among serotype regardless of the year of isolation suggesting the widespread distribution and persistence of *Salmonella* strains in Brazil.

Although the evolutionary relatedness in *S*. *enterica* has been improved in the last decade due broad molecular approach studies^33^, most often, remains difficult to determine when contamination begins^34^ or which isolate is considered a common evolutionary ancestor. In this concern, SNP trees were reconstructed using *Salmonella* isolates (n = 508) retrieved from GenomeTrakr. Ten different clades were identified as shown in Supplementary Fig. S1 and Supplementary Fig. S2. Of these, five were monophyletic [clades-A, B, E, H, J], and five appear to be from novel clades, since our analysis revealed multiple independent lineages of *S*. *enterica* serovars *S*. Infantis [clade-C], *S*. Schwarzengrund [clade-D], *S*. Minnesota [clade-F], *S*. Kentucky [clade-G] and *S*. Brandenburg [clade-I] (Supplementary Fig. S1).

Clade A was composed of 10 MDR *S*. Typhimurium isolates recovered from Brazil. Besides our 7 isolates, 1 isolate was recovered in 2009 from an industrialized product (CFSAN033917), as well as 2 isolates were recovered from swine collected in 2012 (CFSAN068037) and 2015 (UFRGS-SA052). Non-association with international strains was observed. In this regard, most likely this clade is endemic in the Brazilian food sector. Most isolates (n = 13) clustered in clade B were clinical or host-associated recovered in USA (PRJNA230403). These isolates grouped together with *S*. Infantis isolates (SI017 and SI018) from this study. Interestingly, *S*. Infantis SI690 clustered with 2 pan-susceptible *S*. Infantis identified in 2016 by this study, constituting a new clade, named clade C.

All isolates from clade D were MDR *S*. Schwarzengrund recovered from different sources in Brazil. These 21 isolates did not cluster with isolates from other countries. Clade E was mostly constituted by *S*. Minnesota isolated in the United Kingdom (Supplementary Fig. S1). *S*. Minnesota isolates SMi124, SMi160 and SSc153 were clustered with 3 *S*. Minnesota isolated from *Gallus gallus* in Brazil and 38 *S*. Minnesota isolates recovered in UK, all being predominantly found in humans and food (Supplementary Fig. S1). Among *S*. Minnesota, most showed the same resistance profile and carried *qnrB19* and *bla*_CMY-2_ supporting clonal spread of this lineage. On the other hand, *S*. Minnesota SMi294 did not cluster together with the previous clade being nested with another *S*. Minnesota isolate within clade F (Supplementary Fig. S1). Although this isolate carried *qnrB19*, the resistance profile was pan-susceptible. Therefore, the resistance genotype appear to be a determining factor to clustering this isolate outside of the clade E.

Regarding clade G, two pan-susceptible *S*. Kentucky ST198 were nested between each other, which appear not to be related to the highly-drug resistant clade disseminated in Africa^35^.

Clade H, besides our 2 *S*. Newport isolates recovered from chicken carcass, included an isolate from pilgrims (SAMEA2673767) carrying *bla*_CTX-M-2_.

A novel clade (designated I), clustered only two *S*. Brandenburg isolates (Supplementary Fig. S1), both from poultry samples collected from two different geographic locations in Brazil (Table 1). These isolates were multidrug-resistant harboring important resistance genes, including *qnrB19*, *bla*_CMY-2_, *fosA7*, *aac*(*6*′)-*Iaa*, *sul2* and *tetA* (Table 1). Lastly, clade J was composed of 403 *S*. Heidelberg isolates including strains from Brazil, UK and Germany. Of these isolates, 77 were from our *Salmonella* collection (Supplementary Fig. S2). *S*. Heidelberg, currently circulating in Europe, are most likely part of a Brazilian clade that has been the most frequent in Brazil. In this serovar, the presence of the plasmid-mediated *bla*_CMY-2_, readily mobilized, seems to be a major public health issue.

## Discussion

*S*. *enterica* harboring *qnrB19* has become the most common PMQR gene observed in Brazil and has been increasing in the US^36^. In addition to this study describing *qnrB19* identified among 8 serotypes, there are only three additional reports worldwide on *qnrB19*-producing *S*. Infantis^37^ *S*. Heidelberg^38^ and *S*. Newport^39^ isolated from Colombia, Venezuela and Poland, respectively. In this context, this is the first know report of *qnrB19* in *Salmonella* serovars *S*. Schwarzengrund (poultry and environmental), *S*. Infantis (environmental), *S*. Minnesota (poultry), *S*. Brandenburg (poultry), and *S*. Kentucky (poultry), given that *qnrB19* have been reported in Brazil only in *S*. Corvallis^40^, in *E*. *coli*^41,42^ or *K*. *pneumoniae*^43,44^.

Isolates harboring *qnrE1* had not previously been reported for *Salmonella enterica* isolated from poultry and swine. Interestingly, *qnrE1* was reported in *K*. *pneumoniae* from human in Argentina^23^ and in *K*. *pneumoniae* isolated from a parrot in Brazil^24^. In this regard, to our knowledge, this is the first report of *qnrE1* (poultry and swine) in *S*. *enterica* serovar Typhimurium worldwide. These results emphasize the plasticity of this new plasmid and the presence of IS*Ecp1* might be the key vector to silent spread of this resistance gene. These results will aid in the development of mitigation strategies to limit the global distribution of bacteria harboring these genes, since genomic surveillance study allow us to predict, prevent and manage antibiotic resistance and virulence markers in One Health interface, providing substantial evidences that can be implemented in Brazilian food sector.

Two fluoroquinolone-resistant *S*. Infantis isolated from swine were found to be carrying *qnrS1*. Conversely, *qnrS1* in Brazil was associated only with *E*. *coli*^41^, *K*. *pneumoniae*^43,45^ or *Pseudomonas aeruginosa*^46^. In this regard, the presence of *oqxA/oqxB* in swine highlights their importance as emergence of *qnrS1* and *oqxA/oqxB* quinolone resistance genes being a public health concern in swine production chain.

Lastly, the high prevalence of PMQR shown in this study is consistent with those other studies that found *qnrB19* in 2011^40^, 2012^41^, 2013^43^, 2015^44^, 2017^42^, *qnrS1* in 2012^41^, 2013^43^, 2014^45^, 2016^46^ and *qnrE1* in 2017^24^, in Brazil highlighting an urgent need to strengthen surveillance. This finding emphasizes the importance of persistence of quinolone resistance genes emerging in the poultry and swine production chain and calls for action to arrest further transmission and dissemination.

Although ESBL resistance genes are well described within the environment-food-human interface^6,47^, the wide distribution of CTX-M-8, CTX-M-2 and CMY-2 ESBL/pAmpC genes are regarded as a threat to public health, as they are easily transferred horizontally to other foodborne pathogens and commensal bacteria in gut environment^48^. In fact, the high prevalence of ESBLs/pAmpC strains shown in this study is consistent and highlights the endemic occurrence of broad-spectrum cephalosporin strains in South America^49^. In addition, this is the first report in Brazil, identifying *S*. Heidelberg harboring a new gene, *fosA7*, which encodes resistance to fosfomycin [as previously described in *S*. Heidelberg, also isolated from broiler chicken in Canada]^50^.

Out of the nine sequence types found, ST19 was associated with strains isolated from swine and poultry. This ST19 appears to be closely related to the sub-Saharan Africa ST313 clade, which is gastroenteritis-associated and globally distributed^51,52^. However, based on previous investigations^53^, as well as showed in clade A (Supplementary Fig. S1), we suggest that *S*. Typhimurium ST19 strains from Brazil are genetically distinct from those ST19 strains associated with gastroenteritis worldwide, given they did not cluster with international isolates.

Sequence type 32 was associated with strains isolated from swine and chicken cage after cleaning. This ST is highly conserved in *S*. Infantis and has been associated with a clonal dissemination in food sources and human^21,54,55^. Conversely, isolates from poultry and environmental sources were associated with ST96 and ST15. Interestingly, *S*. Schwarzengrund (ST96) was previously associated with ESBL from poultry in Brazil [2013]^56^, carbapenemase resistant KPC-2-producing *S*. Schwarzengrund from human in Argentina [2014]^57^, and most recently with *mcr*-*1*-producing *S*. Schwarzengrund isolated from poultry in Brazil [2018]^58^. In addition, ST15 which had not been previously reported in Brazil has become the most prevalent and relevant serotype in Brazil (manuscript in preparation). Also of interest, STs 548, 198, 45 and 65 were associated with strains isolated from one source (poultry).

In order to support the current knowledge regard the epidemiological distribution of MDR strains between the food-animal-environmental interface, our results provide valuable information related to distribution of multidrug-resistant *S*. *enterica* serovars in food-producing animal settings. In addition to the range of mobile genetic elements identified in our isolate collection, these data provide additional insights into global mobility and genomic plasticity, which contribute to persistence of strains along food chain.

The widespread of multi-drug resistant *S*. *enterica* in poultry and swine production chain is concerning due the potential transmission to human in the end of food chain. Given these isolates are resistant to fluoroquinolones and third-generation cephalosporin raises a particular concern, since these antibiotics are the first choice for the treatment of salmonellosis. While, our results provide additional evidences of the global mobilization of international clones of *S*. *enterica*, over a 16 year-period, continuous surveillance and additional studies in MDR *S*. *enterica* isolated from human, needs to be established as mitigation strategies to limit their global spread.

## Supplementary information

Supplementary Dataset

## References

[1] Tacconelli E (2018). Discovery, research, and development of new antibiotics: the WHO priority list of antibiotic-resistant bacteria and tuberculosis. Lancet Infect Dis.

[2] Jacoby, G. A., Strahilevitz, J. & Hooper, D. C. Plasmid-mediated quinolone resistance. *Microbiol Spectr* **2**, PLAS-0006-2013, 10.1128/microbiolspec.PLAS-0006-2013 (2014). 10.1128/microbiolspec.PLAS-0006-2013 PMC4288778 25584197

[3] Li L (2013). Spread of *oqxAB* in *Salmonella enterica* serotype Typhimurium predominantly by IncHI2 plasmids. J Antimicrob Chemother.

[4] Redgrave LS, Sutton SB, Webber MA, Piddock LJ (2014). Fluoroquinolone resistance: mechanisms, impact on bacteria, and role in evolutionary success. Trends Microbiol.

[5] Li XP (2016). Clonal spread of *mcr*-*1* in PMQR-carrying ST34 *Salmonella* isolates from animals in China. Sci Rep.

[6] Bevan ER, Jones AM, Hawkey PM (2017). Global epidemiology of CTX-M β-lactamases: temporal and geographical shifts in genotype. J Antimicrob Chemother.

[7] Food and Drug Administration. Bacteriological analytical manual. 8^th^ ed. Gaithersburg, Md.: AOAC International (1998).

[8] Grimont PAD, Weil FX. Antigenic formulae of the *Salmonella* serovars. Institut Pasteur, Paris, France, Centre Collaborateur OMS de Référence et de Recherche pour les *Salmonella* 9th ed.; [166 pp.] (2007).

[9] Guibourdenche M (2010). Supplement 2003–2007 (No. 47) to the White-Kauffmann-Le Minor scheme. Res Microbiol.

[10] Clinical and Laboratory Standards Institute (CLSI). Performance standards for antimicrobial susceptibility testing. 27th ed. CLSI supplement M100. CLSI, Wayne, PA (2017).

[11] U S Food and Drug Administration (FDA). The National Antimicrobial Resistance Monitoring System Manual of Laboratory Methods. Retrieved from, https://www.fda.gov/downloads/AnimalVeterinary/SafetyHealth/AntimicrobialResistance/NationalAntimicrobialResistanceMonitoringSystem/UCM453381.pdf (2015).

[12] Monte DF (2019). Genome Sequencing of an *Escherichia coli* Sequence Type 617 Strain Isolated from Beach Ghost Shrimp (*Callichirus major*) from a Heavily Polluted Ecosystem Reveals a Wider Resistome against Heavy Metals and Antibiotics. Microbiol Resour Announc.

[13] Seemann T (2014). Prokka: rapid prokaryotic genome annotation. Bioinformatics.

[14] Page AJ (2015). Roary: rapid large-scale prokaryote pan genome analysis. Bioinformatics.

[15] Hadfield J (2017). Phandango: an interactive viewer for bacterial population genomics. Bioinformatics.

[16] Page AJ (2016). SNP-sites: rapid efficient extraction of SNPs from multi-FASTA alignments. Microb Genom.

[17] Stamatakis A (2014). RAxML version 8: a tool for phylogenetic analysis and post-analysis of large phylogenies. Bioinformatics.

[18] Pattengale ND, Alipour M, Bininda-Emonds OR, Moret BM, Stamatakis A (2010). How many bootstrap replicates are necessary?. J Comput Biol.

[19] Letunic I, Bork P (2016). Interactive tree of life (iTOL) v3: an online tool for the display and annotation of phylogenetic and other trees. Nucleic Acids Res.

[20] Magiorakos AP (2012). Multidrug-resistant, extensively drug-resistant and pandrug-resistant bacteria: an international expert proposal for interim standard definitions for acquired resistance. Clin Microbiol Infect.

[21] Moura Q (2018). Virulent nontyphoidal *Salmonella* producing CTX-M and CMY-2 β-lactamases from livestock, food and human infection, Brazil. Virulence.

[22] Hopkins KL, Davies RH, Threlfall EJ (2005). Mechanisms of quinolone resistance in *Escherichia coli* and *Salmonella*: recent developments. Int J Antimicrob Agents.

[23] Albornoz E (2017). *qnrE1*, a Member of a New Family of Plasmid-Located Quinolone Resistance Genes, Originated from the Chromosome of Enterobacter Species. Antimicrob. Agents Chemother.

[24] Cunha MPV (2017). Complete DNA Sequence of an IncM1 Plasmid Bearing the Novel *qnrE1* Plasmid-Mediated Quinolone Resistance Variant and *bla*(CTX-M-8) from *Klebsiella pneumoniae* Sequence Type 147. Antimicrob Agents Chemother.

[25] Norizuki C (2017). Specific *bla*(CTX-M-8)/IncI1 Plasmid Transfer among Genetically Diverse *Escherichia coli* Isolates between Humans and Chickens. Antimicrob Agents Chemother.

[26] Liu F (2011). Novel virulence gene and clustered regularly interspaced short palindromic repeat (CRISPR) multilocus sequence typing scheme for subtyping of the major serovars of *Salmonella enterica* subsp. *enterica*. Appl Environ Microbiol.

[27] Tang T, Cheng A, Wang M, Li X (2013). Reviews in *Salmonella* Typhimurium PhoP/PhoQ two-component regulatory system. Rev Med Microbiol.

[28] Marcus SL, Brumell JH, Pfeifer CG, Finlay BB (2000). *Salmonella* pathogenicity islands: big virulence in small packages. Microbes Infect.

[29] Abd El Ghany M (2016). Genomic and Phenotypic Analyses Reveal the Emergence of an Atypical *Salmonella enterica* Serovar Senftenberg Variant in China. J Clin Microbiol.

[30] Roer L (2016). Is the Evolution of *Salmonella enterica* subsp. *Enterica* Linked to Restriction-Modification Systems?. mSystems.

[31] Ellis MJ, Trussler RS, Charles O, Haniford DB (2017). A transposon-derived small RNA regulates gene expression in *Salmonella* Typhimurium. Nucleic Acids Res.

[32] Ellis MJ (2018). Silent but deadly: IS200 promotes pathogenicity in *Salmonella* Typhimurium. RNA Biol.

[33] Timme RE (2013). Phylogenetic diversity of the enteric pathogen *Salmonella enterica* subsp. enterica inferred from genome-wide reference-free SNP characters. Genome Biol Evol.

[34] Monte DF, Lincopan N, Fedorka-Cray P, Landgraf M (2019). Current Insights on High Priority Antibiotic-Resistant *Salmonella enterica* in Food and Foodstuffs: A review. Curr Opin Food Sci.

[35] Le Hello S (2013). Highly drug-resistant *Salmonella enterica* serotype Kentucky ST198-X1: a microbiological study. Lancet Infect Dis.

[36] Tyson GH (2017). Identification of Plasmid-Mediated Quinolone Resistance in *Salmonella* Isolated from Swine Ceca and Retail Pork Chops in the United States. Antimicrob Agents Chemother.

[37] Karczmarczyk M (2010). Fanning S. Characterization of antimicrobial resistance in *Salmonella enterica* food and animal isolates from Colombia: identification of a *qnrB19*-mediated quinolone resistance marker in two novel serovars. FEMS Microbiol Lett.

[38] González F, Araque M (2013). Association of Transferable Quinolone Resistance Determinant *qnrB19* with Extended-Spectrum β -Lactamases in *Salmonella* Give and *Salmonella* Heidelberg in Venezuela. Int J Microbiol.

[39] Wasyl D, Hoszowski A, Zając M (2014). Prevalence and characterisation of quinolone resistance mechanisms in *Salmonella* spp. Vet Microbiol.

[40] Ferrari R (2011). Plasmid-mediated quinolone resistance by genes *qnrA1* and *qnrB19* in *Salmonella* strains isolated in Brazil. J Infect Dev Ctries.

[41] Paiva MC, Nascimento AM, Camargo IL, Lima-Bittencourt CI, Nardi RM (2012). The first report of the *qnrB19, qnrS1 and aac*(*6*′)-*Ib*-*cr* genes in urinary isolates of ciprofloxacin-resistant *Escherichia coli* in Brazil. Mem Inst Oswaldo Cruz.

[42] Cunha MP (2017). Coexistence of CTX-M-2, CTX-M-55, CMY-2, FosA3, and QnrB19 in Extraintestinal Pathogenic *Escherichia coli* from Poultry in Brazil. Antimicrob Agents Chemother.

[43] Viana AL (2013). Extended-spectrum β-lactamases in Enterobacteriaceae isolated in Brazil carry distinct types of plasmid-mediated quinolone resistance genes. J Med Microbiol.

[44] Martins WM (2015). Coproduction of KPC-2 and QnrB19 in *Klebsiella pneumoniae* ST340 isolate in Brazil. Diagn Microbiol Infect Dis.

[45] Andrade LN (2014). Expansion and evolution of a virulent, extensively drug-resistant (polymyxin B-resistant), QnrS1-, CTX-M-2-, and KPC-2-producing *Klebsiella pneumoniae* ST11 international high-risk clone. J Clin Microbiol.

[46] Araujo BF (2016). Clinical and Molecular Epidemiology of Multidrug-Resistant *P*. *aeruginosa* Carrying *aac*(*6*′)-*Ib*-*cr*, *qnrS1* and *bla*_SPM_ Genes in Brazil. PLoS One.

[47] Sjölund-Karlsson M (2011). CTX-M-producing non-Typhi *Salmonella* spp. isolated from humans, United States. Emerg Infect Dis.

[48] Card RM (2017). An *In Vitro* Chicken Gut Model Demonstrates Transfer of a Multidrug Resistance Plasmid from *Salmonella* to Commensal *Escherichia coli*. MBio.

[49] Brown AC (2018). CTX-M-65 Extended-Spectrum β-Lactamase-Producing *Salmonella enterica* Serotype Infantis, United States. Emerg Infect Dis.

[50] Rehman MA, Yin X, Persaud-Lachhman MG, Diarra MS (2017). First Detection of a Fosfomycin Resistance Gene, *fosA7*, in *Salmonella enterica* Serovar Heidelberg Isolated from Broiler Chickens. Antimicrob Agents Chemother.

[51] Ramachandran G (2017). Virulence of invasive *Salmonella* Typhimurium ST313 in animal models of infection. PLoS Negl Trop Dis.

[52] Hammarlöf DL (2018). Role of a single noncoding nucleotide in the evolution of an epidemic African clade of *Salmonella*. Proc Natl Acad Sci USA.

[53] Panzenhagen PHN (2018). Genetically distinct lineages of *Salmonella* Typhimurium ST313 and ST19 are present in Brazil. Int J Med Microbiol.

[54] Hauser E (2012). Clonal dissemination of *Salmonella enterica* serovar Infantis in Germany. Foodborne Pathog Dis.

[55] Almeida F, Pitondo-Silva A, Oliveira MA, Falcão JP (2013). Molecular epidemiology and virulence markers of *Salmonella* Infantis isolated over 25 years in São Paulo State, Brazil. Infect Genet Evol.

[56] Silva KC (2013). Emergence of extended-spectrum–lactamase CTX-M-2-producing *Salmonella enterica* serovars Schwarzengrund and Agona in poultry farms. Antimicrob Agents Chemother.

[57] Jure MA (2014). Emergence of KPC-2-Producing *Salmonella enterica* Serotype Schwarzengrund in Argentina. Antimicrob Agents Chemother.

[58] Moreno LZ (2018). First report of *mcr*-*1*-harboring *Salmonella enterica* serovar Schwarzengrund isolated from poultry meat in Brazil. Diagn Microbiol Infect Dis.

