# Supplementary material for: Genomic Features of High-Priority Salmonella enterica Serovars Circulating in the Food Production Chain, Brazil, 2000–2016
Source: Sci Rep. 2019 Jul 30;9:11058. doi: 10.1038/s41598-019-45838-0 (PMC6667439; doi:10.1038/s41598-019-45838-0)
Supplement: Supplementary file 1 — Supplementary Dataset [file 41598_2019_45838_MOESM1_ESM.pdf]

Genomic Features of High-Priority *Salmonella enterica* Serovars Circulating in the Food Production Chain, Brazil, 2000-2016

Daniel F. Monte[1,4*], Nilton Lincopan[2,3], Hanna Berman[4], Louise Cerdeira[3], Shivaramu Keelara[4], Siddhartha Thakur[4], Paula J. Fedorka-Cray[4], and Mariza Landgraf[1]

[1]Department of Food and Experimental Nutrition, Faculty of Pharmaceutical Sciences, Food Research Center, University of São Paulo, Brazil.

[2]Department of Microbiology, Institute of Biomedical Sciences, University of São Paulo, Brazil.

[3]Department of Clinical Analysis, Faculty of Pharmaceutical Sciences, University of São Paulo, Brazil.

[4]Department of Population Health and Pathobiology, North Carolina State University, College of Veterinary Medicine, Raleigh, North Carolina, USA

*Correspondence and requests for materials should be addressed to Daniel F. Monte (email: monte_dfm@usp.br) or Mariza Landgraf (email: landgraf@usp.br).

Supplementary-Table 1. Serotype, year of isolation, state of origin and source of 264 *Salmonella enterica* isolates studied.

Strain ID	Serotype	Year	Location*	Source
STy1	Typhimurium	2000	SC	Slaughterhouse (soybean meal)
STy2	Typhimurium	2000	PR	Pig liver
STy3	Typhimurium	2000	SC	Turkey carcass
STy5	Typhimurium	2012	SC	Slaughterhouse (feed)
STy6	Typhimurium	2012	SC	Mechanically recovered chicken meat
STy8	Typhimurium	2012	SC	Chicken meat
STy9	Typhimurium	2013	SC	Slaughterhouse
STy10	Typhimurium	2012	SC	Slaughterhouse
STy11	Typhimurium	2014	SC	Slaughterhouse
STy12	Typhimurium	2013	SC	Swine stomach
STy13	Typhimurium	2013	PR	Pork
STy14	Typhimurium	2013	PR	Salted chicken breast
STy15	Typhimurium	2014	PR	Mechanically recovered chicken meat
STy16	Typhimurium	2014	PR	Mechanically recovered chicken meat
STy18	Typhimurium	2014	PR	Carcass
STy01	Typhimurium	2015	SP	Broiler chicken
STy03	Typhimurium	2015	SP	Broiler chicken
STy04	Typhimurium	2015	SP	Broiler chicken
STy05	Typhimurium	2015	SP	Broiler chicken
STy06	Typhimurium	2015	SP	Broiler chicken
STy07	Typhimurium	2015	SP	Broiler chicken
STy08	Typhimurium	2015	SP	Broiler chicken
STy011	Typhimurium	2015	SP	Broiler chicken
STy013	Typhimurium	2015	SP	Broiler chicken
STy014	Typhimurium	2015	SP	Broiler chicken
STy015	Typhimurium	2015	SP	Swine stomach
STy017	Typhimurium	2015	SP	Broiler chicken
ST020	Typhimurium	2015	SP	Broiler chicken
SI6	Typhimurium	2012	PR	Chicken meat
SI07	Typhimurium	2015	SP	Broiler chicken
SI010	Typhimurium	2015	SP	Broiler chicken
SI013	Typhimurium	2015	SP	Broiler chicken
SE1	Typhimurium	2012	SC	Salted chicken breast
SE5	Typhimurium	2012	SC	Chicken carcass
SE7	Typhimurium	2013	SC	Chicken heart
SE11	Typhimurium(O5-)	2014	SC	Chicken breast
STy100922373	Typhimurium	2016	SC	Swine slaughterhouse
STy100922376	Typhimurium	2016	SC	Swine slaughterhouse

Supplementary-Table 1. (Continued)

Strain ID	Serotype	Year	Location*	Source
STy9727572	Typhimurium	2016	SC	Swine slaughterhouse
STy9727578	Typhimurium	2016	SC	Swine slaughterhouse
STy8876448	Typhimurium	2016	PR	Broiler chicken
STy9376066	Typhimurium	2016	PR	Broiler chicken
STy280	Typhimurium	2016	SC	Mechanically recovered chicken meat
SI1	Infantis	2000	PR	Mechanically recovered chicken meat
SI2	Infantis	2012	PR	Mechanically recovered chicken meat
SI3	Infantis	2012	PR	Carcass
SI4	Infantis	2012	PR	Slaughterhouse
SI5	Infantis	2012	PR	Mechanically recovered chicken meat
SI7	Infantis	2013	PR	Slaughterhouse
SI8	Infantis	2013	PR	Slaughterhouse
SI9	Infantis	2013	PR	Chicken thigh
SI11	Infantis	2013	PR	Mechanically recovered chicken meat
SI12	Infantis	2013	PR	Chicken breast
SI13	Infantis	2013	PR	Mechanically recovered chicken meat
SI14	Infantis	2014	PR	Slaughterhouse
SI15	Infantis	2014	PR	Chicken thigh
SI16	Infantis	2014	PR	Chicken breast in natura
SI23	Infantis	2014	PR	Chicken meat
SI24	Infantis	2014	PR	Boneless chicken thigh
SI27	Infantis	2014	PR	Chicken meat
SI03	Infantis	2015	SP	Broiler chicken
SI05	Infantis	2015	SP	Broiler chicken
SI08	Infantis	2015	SP	Broiler chicken
SI012	Infantis	2015	SP	Chicken carcass
SI017	Infantis	2015	SP	Swine muscle
SI018	Infantis	2015	SP	Swine muscle
SI019	Infantis	2015	SP	Broiler chicken
SI020	Infantis	2015	SP	Broiler chicken
SI10088955	Infantis	2016	PR	Broiler chicken
SI10124816	Infantis	2016	PR	Broiler chicken
SI625	Infantis	2016	SC	Mechanically recovered chicken meat
SI626	Infantis	2016	SC	Chicken gizzard
SI690	Infantis	2016	SC	Chicken cage after cleaning
SI708	Infantis	2016	SC	Truck after cleaning
SE19	Infantis	2014	SC	Heart
SI10105686	Infantis	2016	MS	Broiler chicken

Supplementary-Table 1. (Continued)

Strain ID	Serotype	Year	Location*	Source
SH498	Infantis	2016	BA	Chicken carcass
SH706	Infantis	2016	SC	Chicken liver
SE2	Enteritidis	2012	SC	Salted chicken breast
SE3	Enteritidis	2012	SC	Chicken wing
SE4	Enteritidis	2012	SC	Salted chicken breast
SE8	Enteritidis	2013	SC	Chicken carcass
SE9	Enteritidis	2014	SC	Mechanically recovered chicken meat
SE10	Enteritidis	2014	SC	Mechanically recovered chicken meat
SE12	Enteritidis	2014	SC	Chicken wing
SE13	Enteritidis	2014	SC	Chicken wing
SE14	Enteritidis	2014	SC	Chicken skin
SE15	Enteritidis	2014	SC	Chicken breast
SE16	Enteritidis	2014	SC	Mechanically recovered chicken meat
SE17	Enteritidis	2014	SC	Salted chicken breast
SE18	Enteritidis	2014	SC	Salted chicken breast
SE20	Enteritidis	2014	SC	Heart
SE03	Enteritidis	2016	SP	Broiler chicken
SE06	Enteritidis	2016	SP	Broiler chicken
SE08	Enteritidis	2016	SP	Broiler chicken
SE09	Enteritidis	2016	SP	Broiler chicken
SE990542	Enteritidis	2016	PR	Broiler chicken
SE10059883	Enteritidis	2016	PR	Broiler chicken
SSc117	Schwarzengrund	2016	SP	Swab
SSc119	Schwarzengrund	2016	SP	Mechanically recovered chicken meat
SSc123	Schwarzengrund	2016	SP	Mechanically recovered chicken meat
SSc126	Schwarzengrund	2016	SP	Chicken thigh
SSc130	Schwarzengrund	2016	SP	Chicken cage after cleaning
SSc140	Schwarzengrund	2016	DF	Chicken wing paddle
SSc146	Schwarzengrund	2016	DF	Chicken carcass
SSc149	Schwarzengrund	2016	DF	Chicken carcass
SSc150	Schwarzengrund	2016	DF	Chicken carcass
SSc151	Schwarzengrund	2016	DF	Chicken carcass
SSc156	Schwarzengrund	2016	DF	Chicken carcass
SSc161	Schwarzengrund	2016	MG	Mechanically recovered chicken meat
SN141	Schwarzengrund	2016	DF	Chicken carcass
SN143	Schwarzengrund	2016	DF	Chicken carcass
SN145	Schwarzengrund	2016	DF	Chicken carcass
SMi132	Schwarzengrund	2016	SP	Chicken carcass

Supplementary-Table 1. (Continued)

Strain ID	Serotype	Year	Location*	Source
SMi152	Schwarzengrund	2016	DF	Chicken carcass
SH137	Schwarzengrund	2016	SP	Chicken liver
SH147	Schwarzengrund	2016	DF	Chicken carcass
SH154	Schwarzengrund	2016	DF	Chicken carcass
SH157	Schwarzengrund	2016	DF	Chicken carcass
SMi124	Minnesota	2016	SP	Chicken carcass
SMi160	Minnesota	2016	MG	Chicken feet
SMi294	Minnesota	2016	SP	Mechanically recovered chicken meat
SMi295	Minnesota	2016	SP	Mechanically recovered chicken meat
SMi416	Minnesota	2016	SC	Slaughterhouse
SSc153	Minnesota	2016	DF	Chicken carcass
SOu718	Ouakan	2016	PR	Chicken carcass
STy4	Havana	2014	SC	Salted chicken breast
SMu162	Muenchen	2016	MG	Chicken breast
SGru165	Grumpensis	2016	SP	Viscera
SI17	Carrau	2014	PR	Swine plasma
SI666	Idikan	2016	SC	Slaughterhouse
SA281	Abony	2016	SC	Slaughterhouse
SA330	Abony	2016	PR	Chicken carcass
SA436	Abony	2016	SC	Chicken pizza
SA438	Abony	2016	SC	Whole lasagna
SH409	Abony	2016	SC	Chicken liver
SH413	Abony	2016	SC	Chicken wing
S260	1,4,[5], 12:i:-	2016	PR	Swine carcass
S262	1,4,[5], 12:i:-	2016	PR	Swine carcass
S417	1,4,[5], 12:i:-	2016	SC	Slaughterhouse
S711	1,4,[5], 12:i:-	2016	SP	Mechanically recovered chicken meat
SH426	1,4,[5], 12:i:-	2016	SC	Truck after cleaning
SIsa406	Isangi	2016	SC	Slaughterhouse
SIsa424	Isangi	2016	SC	Slaughterhouse
SIsa425	Isangi	2016	SC	Slaughterhouse
SI667	Isangi	2016	SC	Slaughterhouse
SSe331	Senftenberg	2016	PR	Chicken carcass
SSe713	Senftenberg	2016	SP	Mechanically recovered chicken meat
SSe717	Senftenberg	2016	PR	Chicken carcass
SH282	Senftenberg	2016	SP	Chicken carcass
SH420	Rissen	2016	SC	Chicken breast fillet
SH421	Rissen	2016	SC	Chicken wing

Supplementary-Table 1. (Continued)

Strain ID	Serotype	Year	Location*	Source
SH432	Rissen	2016	SC	Chicken liver
SH10230048	Rissen	2016	RS	Broiler chicken
SOH293	Ohio	2016	SP	Viscera
SOH813	Ohio	2016	RS	Feather meal
SI10	Ohio	2013	PR	Viscera
ST010	Ohio	2015	SP	Broiler chicken
SI02	Mbandaka	2015	SP	Broiler chicken
SI22	Mbandaka	2014	PR	Chicken carcass
SMb279	Mbandaka	2016	SC	Truck after cleaning
STy02	Rochdale	2015	SP	Broiler chicken
SI016	Rochdale	2015	SP	Broiler chicken
SH133	Rochdale	2016	SP	Mechanically recovered chicken meat
SK496	Kentucky	2016	BA	Chicken thigh and drumstick
SK497	Kentucky	2016	BA	Chicken liver
SSc142	Newport	2016	DF	Chicken carcass
SN144	Newport	2016	DF	Chicken carcass
SH291	Brandenburg	2016	SP	Chicken breast
SH686	Brandenburg	2016	SC	Mechanically recovered chicken meat
SI18	Saphra	2014	PR	Chicken meat
SI19	Saphra	2014	PR	Boneless chicken thigh
SMon163	Montevideo	2016	MG	Feather meal
STy09	Montevideo	2015	SP	Broiler chicken
SI25	Panama	2014	PR	Mechanically recovered chicken meat
SP263	Panama	2016	PR	Swine tongue
SOr259	Orion	2016	PR	Chicken carcass
SH720	Orion	2016	PR	Chicken carcass
SH1	Heidelberg	2016	SP	Mechanically recovered chicken meat
SH018	Heidelberg	2016	SP	Broiler chicken
SH019	Heidelberg	2016	SP	Broiler chicken
SH118	Heidelberg	2016	SP	Chicken breast
SH120	Heidelberg	2016	SP	Chicken thigh and drumstick
SH121	Heidelberg	2016	SP	Chicken fillet sassami
SH122	Heidelberg	2016	SP	Chicken carcass
SH125	Heidelberg	2016	SP	Chicken carcass
SH127	Heidelberg	2016	SP	Chicken fillet sassami
SH128	Heidelberg	2016	SP	Chicken carcass
SH129	Heidelberg	2016	SP	Chicken carcass
SH131	Heidelberg	2016	SP	Mechanically recovered chicken meat

Supplementary-Table 1. (Continued)

Strain ID	Serotype	Year	Location*	Source
SH134	Heidelberg	2016	SP	Chicken cage after cleaning
SH135	Heidelberg	2016	SP	Whole chicken
SH138	Heidelberg	2016	SP	Leg quarter
SH158	Heidelberg	2016	MG	Fiesta boneless
SH159	Heidelberg	2016	MG	Chicken cage after cleaning
SH164	Heidelberg	2016	SP	Viscera
SH258	Heidelberg	2016	PR	Chicken carcass
SH264	Heidelberg	2016	PR	Truck after cleaning
SH265	Heidelberg	2016	PR	Truck after cleaning
SH266	Heidelberg	2016	PR	Chicken carcass
SH268	Heidelberg	2016	PR	Carcass after chiller
SH269	Heidelberg	2016	PR	Chicken carcass after chiller
SH270	Heidelberg	2016	PR	Chicken carcass after chiller
SH276	Heidelberg	2016	SP	Salted chicken breast
SH283	Heidelberg	2016	SP	Chicken carcass
SH284	Heidelberg	2016	SP	Chicken carcass
SH285	Heidelberg	2016	SP	Chicken carcass
SH286	Heidelberg	2016	SP	Chicken thigh and drumstick
SH287	Heidelberg	2016	SP	Chicken skin
SH289	Heidelberg	2016	SP	Seasoned chicken fillet
SH290	Heidelberg	2016	SP	Chicken liver
SH296	Heidelberg	2016	SP	Mechanically recovered chicken meat
SH297	Heidelberg	2016	SP	Mechanically recovered chicken meat
SH402	Heidelberg	2016	SC	Chicken liver
SH403	Heidelberg	2016	SC	Seasoned chicken fillet
SH405	Heidelberg	2016	SC	Chicken breast fillet
SH408	Heidelberg	2016	SC	Chicken liver
SH410	Heidelberg	2016	SC	Chicken breast fillet
SH411	Heidelberg	2016	SC	Chicken thigh and drumstick
SH412	Heidelberg	2016	SC	Chicken wing
SH414	Heidelberg	2016	SC	Truck after cleaning
SH415	Heidelberg	2016	SC	Chicken cage after cleaning
SH422	Heidelberg	2016	SC	Chicken liver
SH423	Heidelberg	2016	SC	Chicken liver
SH427	Heidelberg	2016	SC	Whole chicken
SH429	Heidelberg	2016	SC	Chicken liver
SH430	Heidelberg	2016	SC	Chicken liver
SH431	Heidelberg	2016	SC	Chicken liver

Supplementary-Table 1. (Continued)

Strain ID	Serotype	Year	Location*	Source
SH433	Heidelberg	2016	SC	Truck after cleaning
SH434	Heidelberg	2016	SC	Chicken cage after cleaning
SH435	Heidelberg	2016	SC	Truck after cleaning
SH674	Heidelberg	2016	SC	Chicken liver
SH680	Heidelberg	2016	SC	Retail meat
SH681	Heidelberg	2016	SC	Chicken wing
SH685	Heidelberg	2016	SC	Chicken neck
SH687	Heidelberg	2016	SC	Chicken liver
SH691	Heidelberg	2016	SC	Chicken wing
SH692	Heidelberg	2016	SC	Chicken thigh and drumstick
SH693	Heidelberg	2016	SC	Chicken wing
SH694	Heidelberg	2016	SC	Chicken breast fillet
SH697	Heidelberg	2016	SC	Mechanically recovered chicken meat
SH700	Heidelberg	2016	SC	Mechanically recovered chicken meat
SH707	Heidelberg	2016	SC	Chicken liver
SH712	Heidelberg	2016	SP	Mechanically recovered chicken meat
SH715	Heidelberg	2016	SP	Chicken cage after cleaning
SH716	Heidelberg	2016	SP	Chicken Wing Paddle
SH10211124	Heidelberg	2016	SC	Broiler chicken
SH10227492	Heidelberg	2016	SC	Broiler chicken
SH10230633	Heidelberg	2016	MS	Broiler chicken
SH10190712	Heidelberg	2016	PR	Broiler chicken
SH10201911	Heidelberg	2016	SC	Broiler chicken
SH10206799	Heidelberg	2016	SC	Broiler chicken
10225532	Heidelberg	2016	SC	Broiler chicken
STy012	Heidelberg	2015	SP	Broiler chicken
SI015	Heidelberg	2015	SP	Broiler chicken
SSc136	Heidelberg	2016	SP	Chicken Wing Paddle
SSc139	Heidelberg	2016	SP	Chicken wing
SSc148	Heidelberg	2016	DF	Chicken carcass
SSc155	Heidelberg	2016	DF	Chicken carcass

*DF: Distrito Federal; MG: Minas Gerais; SP: São Paulo; PR: Paraná; SC: Santa Catarina, BA: Bahia.

Supplementary Figure legends

Supplementary Fig. S1. SNP based phylogram of 105 *S. enterica* serovars isolates from various isolation sources and locations. Strain ID, isolate source, location, SNP cluster, collection date and serovars were retrieved from Genebank. Red color indicates isolates from this study.

Supplementary Fig. S2. SNP based phylogram of 403 *S. enterica* serovar Heidelberg isolates from various isolation sources and locations. Strain ID, isolate source, location, SNP cluster, collection date and serovars were retrieved from Genebank. Red color indicates isolates from this study.

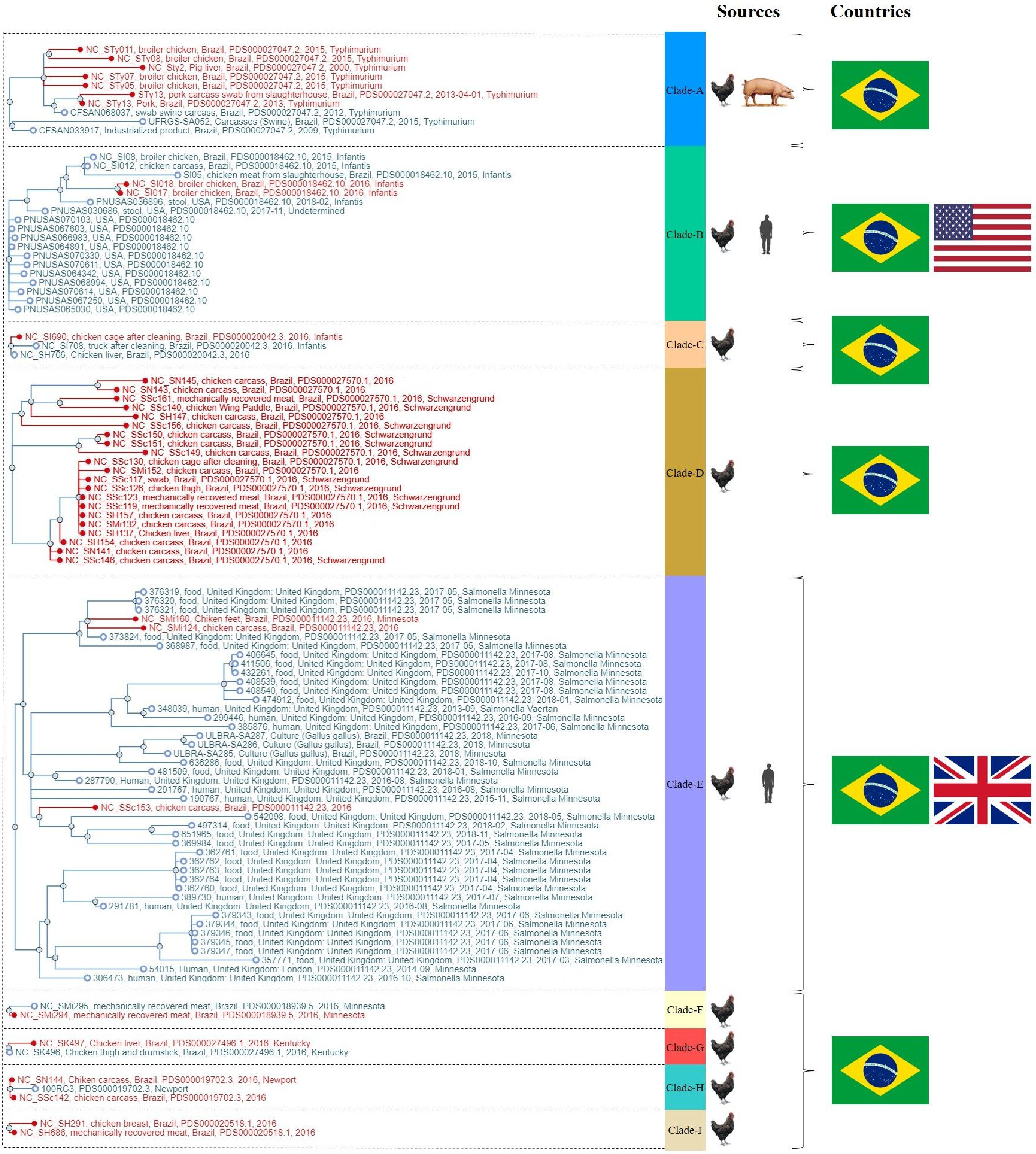

Sources
Countries
NC_STy011, broiler chicken, Brazil, PDS000027047.2, 2015, Typhimurium
NC_STy08, broiler chicken, Brazil, PDS000027047.2, 2015, Typhimurium
NC_Sty2, Pig liver, Brazil, PDS000027047.2, 2000, Typhimurium
NC_STy07, broiler chicken, Brazil, PDS000027047.2, 2015, Typhimurium
NC_STy05, broiler chicken, Brazil, PDS000027047.2, 2015, Typhimurium
STy13, pork carcass swab from slaughterhouse, Brazil, PDS000027047.2, 2013-04-01, Typhimurium
NC_STy13, Pork, Brazil, PDS000027047.2, 2013, Typhimurium
CFSAN068037, swab swine carcass, Brazil, PDS000027047.2, 2012, Typhimurium
UFRGS-SA052, Carcasses (Swine), Brazil, PDS000027047.2, 2015, Typhimurium
CFSAN033917, Industrialized product, Brazil, PDS000027047.2, 2009, Typhimurium
Clade-A
NC_SI08, broiler chicken, Brazil, PDS000018462.10, 2015, Infantis
NC_SI012, chicken carcass, Brazil, PDS000018462.10, 2015, Infantis
SI05, chicken meat from slaughterhouse, Brazil, PDS000018462.10, 2015, Infantis
NC_SI018, broiler chicken, Brazil, PDS000018462.10, 2016, Infantis
NC_SI017, broiler chicken, Brazil, PDS000018462.10, 2016, Infantis
PNUSAS036896, stool, USA, PDS000018462.10, 2018-02, Infantis
PNUSAS030686, stool, USA, PDS000018462.10, 2017-11, Undetermined
PNUSAS070103, USA, PDS000018462.10
PNUSAS067603, USA, PDS000018462.10
PNUSAS066983, USA, PDS000018462.10
PNUSAS064891, USA, PDS000018462.10
PNUSAS070330, USA, PDS000018462.10
PNUSAS070611, USA, PDS000018462.10
PNUSAS064342, USA, PDS000018462.10
PNUSAS068994, USA, PDS000018462.10
PNUSAS070614, USA, PDS000018462.10
PNUSAS067250, USA, PDS000018462.10
PNUSAS065030, USA, PDS000018462.10
Clade-B
NC_SI690, chicken cage after cleaning, Brazil, PDS000020042.3, 2016, Infantis
NC_SI708, truck after cleaning, Brazil, PDS000020042.3, 2016, Infantis
NC_SH706, Chicken liver, Brazil, PDS000020042.3, 2016
Clade-C
NC_SN145, chicken carcass, Brazil, PDS000027570.1, 2016
NC_SN143, chicken carcass, Brazil, PDS000027570.1, 2016
NC_SSc161, mechanically recovered meat, Brazil, PDS000027570.1, 2016, Schwarzengrund
NC_SSc140, chicken Wing Paddle, Brazil, PDS000027570.1, 2016, Schwarzengrund
NC_SH147, chicken carcass, Brazil, PDS000027570.1, 2016
NC_SSc156, chicken carcass, Brazil, PDS000027570.1, 2016, Schwarzengrund
NC_SSc150, chicken carcass, Brazil, PDS000027570.1, 2016, Schwarzengrund
NC_SSc151, chicken carcass, Brazil, PDS000027570.1, 2016, Schwarzengrund
NC_SSc149, chicken carcass, Brazil, PDS000027570.1, 2016, Schwarzengrund
NC_SSc130, chicken cage after cleaning, Brazil, PDS000027570.1, 2016, Schwarzengrund
NC_SMi152, chicken carcass, Brazil, PDS000027570.1, 2016
NC_SSc117, swab, Brazil, PDS000027570.1, 2016, Schwarzengrund
NC_SSc126, chicken thigh, Brazil, PDS000027570.1, 2016, Schwarzengrund
NC_SSc123, mechanically recovered meat, Brazil, PDS000027570.1, 2016, Schwarzengrund
NC_SSc119, mechanically recovered meat, Brazil, PDS000027570.1, 2016, Schwarzengrund
NC_SH157, chicken carcass, Brazil, PDS000027570.1, 2016
NC_SMi132, chicken carcass, Brazil, PDS000027570.1, 2016
NC_SH137, Chicken liver, Brazil, PDS000027570.1, 2016
NC_SH154, chicken carcass, Brazil, PDS000027570.1, 2016
NC_SN141, chicken carcass, Brazil, PDS000027570.1, 2016
NC_SSc146, chicken carcass, Brazil, PDS000027570.1, 2016, Schwarzengrund
Clade-D
376319, food, United Kingdom: United Kingdom, PDS000011142.23, 2017-05, Salmonella Minnesota
376320, food, United Kingdom: United Kingdom, PDS000011142.23, 2017-05, Salmonella Minnesota
376321, food, United Kingdom: United Kingdom, PDS000011142.23, 2017-05, Salmonella Minnesota
NC_SMi160, Chiken feet, Brazil, PDS000011142.23, 2016, Minnesota
NC_SMi124, chicken carcass, Brazil, PDS000011142.23, 2016
373824, food, United Kingdom: United Kingdom, PDS000011142.23, 2017-05, Salmonella Minnesota
368987, food, United Kingdom: United Kingdom, PDS000011142.23, 2017-05, Salmonella Minnesota
406645, food, United Kingdom: United Kingdom, PDS000011142.23, 2017-08, Salmonella Minnesota
411506, food, United Kingdom: United Kingdom, PDS000011142.23, 2017-08, Salmonella Minnesota
432261, food, United Kingdom: United Kingdom, PDS000011142.23, 2017-10, Salmonella Minnesota
408539, food, United Kingdom: United Kingdom, PDS000011142.23, 2017-08, Salmonella Minnesota
408540, food, United Kingdom: United Kingdom, PDS000011142.23, 2017-08, Salmonella Minnesota
474912, food, United Kingdom: United Kingdom, PDS000011142.23, 2018-01, Salmonella Minnesota
348039, human, United Kingdom: United Kingdom, PDS000011142.23, 2013-09, Salmonella Vaertan
299446, human, United Kingdom: United Kingdom, PDS000011142.23, 2016-09, Salmonella Minnesota
385876, human, United Kingdom: United Kingdom, PDS000011142.23, 2017-06, Salmonella Minnesota
ULBRA-SA287, Culture (Gallus gallus), Brazil, PDS000011142.23, 2018, Minnesota
ULBRA-SA286, Culture (Gallus gallus), Brazil, PDS000011142.23, 2018, Minnesota
ULBRA-SA285, Culture (Gallus gallus), Brazil, PDS000011142.23, 2018, Minnesota
636286, food, United Kingdom: United Kingdom, PDS000011142.23, 2018-10, Salmonella Minnesota
481509, food, United Kingdom: United Kingdom, PDS000011142.23, 2018-01, Salmonella Minnesota
287790, Human, United Kingdom: United Kingdom, PDS000011142.23, 2016-08, Salmonella Minnesota
291767, human, United Kingdom: United Kingdom, PDS000011142.23, 2016-08, Salmonella Minnesota
190767, human, United Kingdom: United Kingdom, PDS000011142.23, 2015-11, Salmonella Minnesota
NC_SSc153, chicken carcass, Brazil, PDS000011142.23, 2016
542098, food, United Kingdom: United Kingdom, PDS000011142.23, 2018-05, Salmonella Minnesota
497314, food, United Kingdom: United Kingdom, PDS000011142.23, 2018-02, Salmonella Minnesota
651965, food, United Kingdom: United Kingdom, PDS000011142.23, 2018-11, Salmonella Minnesota
369984, food, United Kingdom: United Kingdom, PDS000011142.23, 2017-05, Salmonella Minnesota
362761, food, United Kingdom: United Kingdom, PDS000011142.23, 2017-04, Salmonella Minnesota
362762, food, United Kingdom: United Kingdom, PDS000011142.23, 2017-04, Salmonella Minnesota
362763, food, United Kingdom: United Kingdom, PDS000011142.23, 2017-04, Salmonella Minnesota
362764, food, United Kingdom: United Kingdom, PDS000011142.23, 2017-04, Salmonella Minnesota
362760, food, United Kingdom: United Kingdom, PDS000011142.23, 2017-04, Salmonella Minnesota
389730, human, United Kingdom: United Kingdom, PDS000011142.23, 2017-07, Salmonella Minnesota
291781, human, United Kingdom: United Kingdom, PDS000011142.23, 2016-08, Salmonella Minnesota
379343, food, United Kingdom: United Kingdom, PDS000011142.23, 2017-06, Salmonella Minnesota
379344, food, United Kingdom: United Kingdom, PDS000011142.23, 2017-06, Salmonella Minnesota
379346, food, United Kingdom: United Kingdom, PDS000011142.23, 2017-06, Salmonella Minnesota
379345, food, United Kingdom: United Kingdom, PDS000011142.23, 2017-06, Salmonella Minnesota
379347, food, United Kingdom: United Kingdom, PDS000011142.23, 2017-06, Salmonella Minnesota
357771, food, United Kingdom: United Kingdom, PDS000011142.23, 2017-03, Salmonella Minnesota
54015, Human, United Kingdom: London, PDS000011142.23, 2014-09, Minnesota
306473, human, United Kingdom: United Kingdom, PDS000011142.23, 2016-10, Salmonella Minnesota
Clade-E
NC_SMi295, mechanically recovered meat, Brazil, PDS000018939.5, 2016, Minnesota
NC_SMi294, mechanically recovered meat, Brazil, PDS000018939.5, 2016, Minnesota
Clade-F
NC_SK497, Chicken liver, Brazil, PDS000027496.1, 2016, Kentucky
NC_SK496, Chicken thigh and drumstick, Brazil, PDS000027496.1, 2016, Kentucky
Clade-G
NC_SN144, Chiken carcass, Brazil, PDS000019702.3, 2016, Newport
100RC3, PDS000019702.3, Newport
NC_SSc142, chicken carcass, Brazil, PDS000019702.3, 2016
Clade-H
NC_SH291, chicken breast, Brazil, PDS000020518.1, 2016
NC_SH686, mechanically recovered meat, Brazil, PDS000020518.1, 2016
Clade-I

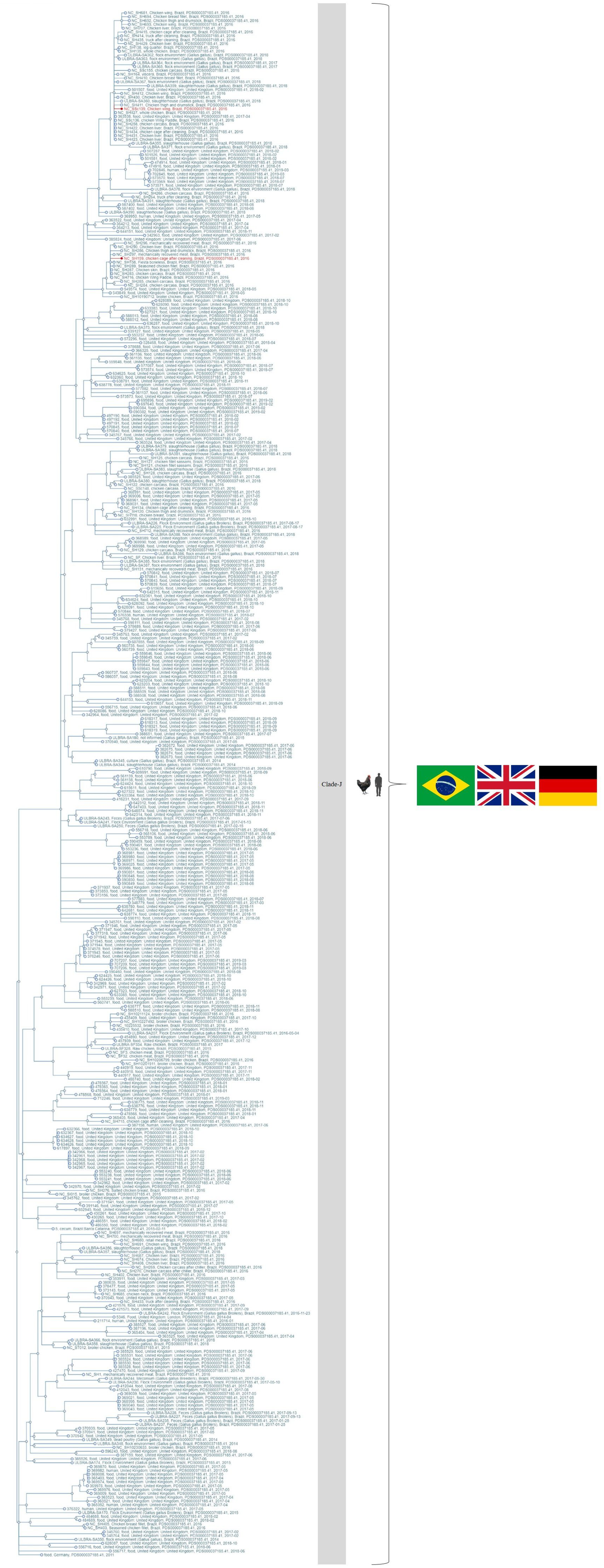
Clade-J
